# Plant Feed Additives as Natural Alternatives to the Use of Synthetic Antioxidant Vitamins on Poultry Performances, Health, and Oxidative Status: A Review of the Literature in the Last 20 Years

**DOI:** 10.3390/antiox10050659

**Published:** 2021-04-23

**Authors:** Federico Righi, Rosario Pitino, Carmen L. Manuelian, Marica Simoni, Afro Quarantelli, Massimo De Marchi, Eleni Tsiplakou

**Affiliations:** 1 Department of Veterinary Science, University of Parma, Via del Taglio 10, 43126 Parma, Italy; rosario.pitino@unipr.it (R.P.); marica.simoni@unipr.it (M.S.); afro.quarantelli@unipr.it (A.Q.); 2 Department of Agronomy, Food, Natural Resources, Animals and Environment, University of Padova, Viale dell’ Università 16, 35020 Legnaro, Italy; carmenloreto.manuelianfuste@unipd.it (C.L.M.); massimo.demarchi@unipd.it (M.D.M.); 3 Laboratory of Nutritional Physiology and Feeding, Department of Animal Science, School of Animal Biosciences, Agricultural University of Athens, Iera Odos 75, 11855 Athens, Greece

**Keywords:** plant extract, essential oils, plant by-product, natural vitamins, synthetic vitamins, vitamin E, vitamin C, tocopheryl, tocopherols, antioxidants, poultry

## Abstract

Plant feed additives (PFA) such as essential oils, extracts, and by-products from plant processing can be included in poultry diets. A total of 39 peer-reviewed articles were selected from the literature published in the last 20 years (2000–2020) comparing PFA antioxidant effects with synthetic antioxidant vitamins (mainly vitamin E) in poultry nutrition. The PFA can be used as an effective nutritional strategy to face poultry’s oxidative stress with positive impact also on their productivity and efficiency. They can partially or completely replace antioxidant synthetic vitamins (the latter administered at doses between 150 and 500 mg/kg) in animal diets, sometimes affecting important physiological functions or expressing synergistic effect with the synthetic antioxidants. It is crucial to take into consideration the issues related to the absorption and the metabolism of these additives and their interaction with gut microbiota. However, some form- and dose-dependent negative effects on growth performances are observed.

## 1. Introduction

Plant feed additives (PFA) constitute a wide group of biologically active compounds with potential positive effects on animal health and productivity [1,2]. The antioxidant components of PFA belong to different chemical classes that can be recovered and/or obtained as plant extracts, essential oils, or resins [3]. Compounds in PFA are generally plant secondary metabolites resulting from a long adaptation process of plants to face infections and environmental stressors [4]. It is possible to isolate polyphenols, which include flavonoids, phenolic acids, and stillbenoids, from PFA [5]. Within polyphenols, flavonoids are the more abundant group and comprehend several compounds of interest for animal feeding.

Essential oils are mixtures of different volatile and non-volatile compounds, chemically divided in two main groups: terpenoids (e.g., thymol, carvacrol) and phenylpropanoids (e.g., cinnamaldehyde, eugenol) [6]. Some of these molecules have a synergistic role in the antioxidant system. For instance, the antioxidant properties of rosemary as a whole plant is due to the simultaneous presence of phenolic diterpenes, phenolic acids (polyphenols), and essential oils, showing the capacity of scavenging free radicals as vitamin E (VitE) and C (VitC) [3]. Similarly, by-products from grape industry as well as from citrus contain a wide range of polyphenols [7,8], such as epicatechin, hesperidin, and quercetin, with the highest antioxidant potential [9].

Vitamins are nutrient compounds needed in small amounts (mg per day) which are essential for many metabolic processes and for the efficient use of other nutrients. They are naturally present in animals’ ration ingredients, but their supplementation is needed to guarantee the fulfillment of the animals’ requirements. VitE and VitC are considered exogenous antioxidants. In fact, they play a key role in the animal’s oxidative stress balance, as it was widely reviewed in poultry. For example, VitE has an essential role in stabilizing the cellular membranes [5].

Since use of antibiotics as growth promoters was banned in the EU for poultry production in 2006, several management and nutritional strategies in poultry industry were proposed in order to maintain high standards of productivity, healthiness, and welfare [6,9,10,11,12]. Among the nutritional strategies, the antioxidant activity of PFA compared with synthetic vitamins was assessed [9]. Under this scenario, several studies evaluated the potential of PFA and plant industry by-products as alternatives to vitamin in poultry nutrition. Nevertheless, the exploitation of nutritional strategies based on plant origin components is still ongoing due to their many challenges: control of zoonotic diseases of public relevance, sustainability, quality of products, perception of consumer, and growing restraints on synthetic antimicrobial use for therapeutic purposes [12,13].

Therefore, a systematic review including peer-reviewed papers published between 2000 and 2020 was conducted regarding the antioxidant capacity of plant extract (PE), essential oils (EO), and by-products of plant origin (BP) as potential alternatives to synthetic vitamins in livestock and the effect of PFA on animals’ performance and physiological parameters compared to synthetic antioxidants.

## 2. Materials and Methods

A systematic review of peer-reviewed papers published in Pubmed (www.ncbi.nlm.nih.gov; last accessed on 10 January 2021), ISI Web of Science (www.webofknowledge.com; last accessed on 10 January 2021), and Science direct (www.sciencedirect.com; last accessed on 10 January 2021) databases was performed covering a time-span of 20 years (January 2000 to December 2020). Studies were selected if they reported: (i) comparisons of the effects either of PE, EO, or BP from different agro-industries with a specific dose of synthetic antioxidant vitamins—or antioxidants in general—in poultry nutrition and (ii) the use of additives of plant origin (excluded propolis and algae). A total of 39 papers from 14 different countries fulfilled both criteria and were included in the present review. A total of 20.5% of the selected papers were from Greece, based on the country from the corresponding authors. Most papers evaluated the effect of PE or EO (*n* = 31) on poultry, and fewer focused on the BP (*n* = 8). Most investigated PFAs were essential oils and extracts of rosemary and oregano (*n* = 14) and grape by-products (*n* = 7).

The present review is divided in three sections according to the group of parameters investigated: (i) effects on feed intake, feed efficiency, growth traits, and nutrients digestibility; (ii) effects on metabolic, hematological parameters, and antioxidant status; and (iii) effects on macro- and microscopic anatomical features and on intestinal microbiome profile.

## 3. Potential Plant Extracts and Plant By-Products as Alternative Sources of Vitamins for Poultry Feeding

### 3.1. Effects on Feed Intake, Feed Efficiency, Growth Traits, and Nutrients Digestibility (Table S1 in Supplementary Materials)

#### 3.1.1. Feed Intake

The dietary supplementation of broilers with grape pomace (GP; 0.5% to 6% of the diet) in comparison with VitE (200 mg/kg of the diet) or a basal diet (no VitE added) for 21 days did not affect broilers’ feed intake (FI) [14,15]. Similarly, the dietary inclusion of fermented and unfermented grape skin (FGS and UGS, respectively; 6% of the diet) for 21 days did not affect broilers’ average daily feed intake (ADFI) in comparison with a negative (no VitE) and a positive (200 mg/kg of VitE) control [16]. Similar results were found when comparing VitE (200 mg/kg) with antioxidant compounds (2.5% of rosehip, chokeberry pomace, or nettle separately) supplementation for 28 days [17] or rosemary oil (RO; 0.010%, 0.015%, and 0.020% of the diet) supplementation for 42 days [18] or feeding rosemary leaves (RL; 2.5% of the diet) for 28 days [17]. The FI was also not affected when coneflower extract (CE, 0.056% of the diet), thyme extract (TE, 0.056% of the diet), sage extract (SE, 0.056% of the diet), marigold xanthophyll (MX, 0.02% of the diet), synthetic antioxidants (0.00486% of the diet), or β-apo-8-carotenoic acid ethylester (0.004% of the diet) were separately administered compared with VitE (150 mg/kg) during a 20 day trial [19]. When SE was added to laying hens diet (2.5% of the diet), no significant effects on FI compared to control (deficient VitE diet) or to VitE groups (30 mg/kg of the diet) were reported [20]. The dietary supplementation with oregano oil (OO; 0.01% [21,22] and 0.02% of the diet [21]) compared with VitE (included at 200 mg/kg [21] and 10 or 100 mg/kg [22] of the diet) for 42 days did not affect broilers’ FI. The inclusion of dehydrated oregano plant (OP; 0.5% and 1%) in addition to VitE (170 mg/kg) for 28 days compared with VitE (170 mg/kg) or antibiotic (Flavomycin, 0.0004% of the diet; and Lasalocid, 0.0075% of the diet) enriched diets also showed no effect in turkeys [23]. Supplementation with different levels of RO (0.015% or 0.030% of the diet) or a combination of RO with OO (both at 0.0075% and 0.0150% of the diet, respectively) during a 42 day trial did not affect broilers’ FI compared with VitE (200 mg/kg) [24]. In addition, no significant variations in FI were reported when broilers were supplemented for 42 days with OO, RO, or fennel volatile oil (FVO) (each at 0.01% of the diet) or with a mixture of them (VOM, 0.01 to 0.4%) [25]. Moreover, olive leaf extract (OLE) dietary supplementation (0.2% and 0.4% of the diet) did not affect FI in broilers reared under heat stress (HS) during a 14 day period compared with control and VitE treatments [26].

On the contrary, broilers fed thyme oil (TO; 0.01% and 0.02% of the diet) exhibited a higher FI (by 3.7% on average) compared with those receiving only a basal diet (in which VitE -40 mg/kg- was included in a premix). A similar increase in FI (2.0% on average) was observed in comparison with the VitE (200 mg/kg) supplemented group [27]. Nevertheless, in broilers, the dietary inclusion of rosemary powder (RPO; 1% of the diet) compared with VitE (200 mg/kg) lowered the FI (by 4.3%) in a 42 days feeding trial [28].

In laying hens, the dietary addition of rosemary plant (RP; 0.5% and 1% of the diet) [29,30], OP (0.5% of the diet) [29], or saffron plant (SP; 2% of the diet) [29] compared with VitE (200 mg/kg) did not modify FI. Moreover, Roselle calyx (*Hibiscus sabdariffa* Linn.) powder (RCP; 2% and 4% of the diet) or its extract (RCE; 1% and 2% of the diet) did not affect FI in laying hens throughout an 8 weeks trial compared with VitE (250 mg/kg) [31]. No effect was found on FI between anise seed (AS; 1% and 2% of the diet) and VitE (30, 600, or 300 mg/kg of diet) fed Japanese quails [32]. Only a study in laying hens demonstrated a reduction of FI (by 6%) when green tea extract (GTE; 1.5% of the diet) compared with VitE (200 mg/kg) was administered [33].

In broilers, average daily feed intake (ADFI) did not change when GP (from 0.5% to 10% of the diet) was used for 21 [14,15] or 42 [34] days compared with VitE (200 mg/kg) or negative control (95.3 mg/kg of VitE) groups. No differences were found by adding either a commercial preparation of essential oils (0.05% and 0.1%) or VitE (200 mg/kg) compared with the negative control treatment (only vitamin premix) during a 42 days trial [35]. Conversely, ADFI in broilers tended to be higher when they consumed *Forsythia suspensa* extract (FSE; 100 mg/kg), basal (by 3.6%), or vitamin C (VitC, 200 mg/kg; by 0.9%) diets for 42 days under HS (34 °C) conditions [36].

Feeding broilers under HS conditions (34 °C) with polyphenols (PP), alone (0.02%) or in combination with VitE (PPE, 0.01% PP and 100 mg/kg of VitE) in comparison with two levels of VitE (100 and 200 mg/kg) did not affect FI [37]. However, after 28 days of trial, the same parameter was improved by the dietary addition of PP (of about 19%) compared with VitE (10.60 mg/kg). In two similar studies, PP supplementation did not modify FI in broilers challenged with Ochratoxin A [38] or with low quality oil [39].

In general, all the reported studies in poultry seem to confirm that the dietary supplementation with different PFA does not exert detrimental effects on FI and ADFI when compared with synthetic vitamins or negative control treatments (basal levels of antioxidant vitamins in the vitamin premix). Among these, effects of PFA as growth promoters were widely investigated in different trials in livestock. In our literature review, few studies reported positive (TO in broilers and *Forsythia suspensa* in the same animals under HS) or negative (RPO in broilers and GTE in laying hens) effects of PFA on FI in poultry. In fact, most of them did not report any significant effect compared with basal diet or in comparison with VitE enriched diet. However, for those studies reporting some effects on this parameter, the variability could be related to the dose employed and to dietary and environmental conditions, which vary across the different trials.

#### 3.1.2. Body Weight and Body Weight Gain

An increase (by 10%) in body weight (BW) of broilers under HS (34 °C) was found when their diet was supplemented with grape seed extract (GSE; 0.03% of the diet) compared with VitC (0 or 300 mg/kg) for 42 days [40]. This improvement was half in the first 28 days of trial when HS was still not present. However, GSE administration (2.6% or 5.2% of the diet) alone or in combination with methionine (0.15% of the diet) during a 3 week trial suggested a dose-dependent detrimental effect on average final weight (AFW) and weight gain (WG) compared with the basal and VitE (335 mg/kg) diets [41]. The negative effect on WG was confirmed by other studies which used grape by-products at similar doses (0.03% to 5.2% of the diet) [14,15,34]. When broilers were fed a diet containing FGS and UGS (both at 6% of the diet) for 21 days, the average daily gain (ADG) was negatively affected compared with the control group (by 10.9% on average) and the group supplemented with VitE (200 mg/kg; by 14.43% on average) [16].

The FSE supplementation (0.01% of the diet) during a 42 day trial mitigated the detrimental effect of HS (32 °C) on ADG compared with basal and VitC administration (by 6.5% on average) [36]. Broilers fed a diet including RO (0.01%, 0.015%, and 0.02% of the diet) showed on average greater live WG than animals receiving VitE (by 4.9%) or RL (by 5.2%) after 21 days [18]. Similarly, broilers fed for 21 days with oregano aqueous extract (OAE: 0.002% of the diet) in comparison with control and VitE ones (150 mg/kg) improved (by 8.1% and 11.5%, respectively) their BW [42]. The dietary inclusion of OAE (0.02% of the diet) improved (by an average of 8.1%) broilers’ BW at 21 days of age compared with VitE (150 mg/kg) and at 42 days of age when compared with the control group (by 8.9%). In addition, ADG was only positively affected (by 14.7%) in the 21–42 days interval of treatment and in comparison with the negative control group [43].

The dietary supplementation with OP (0.05% of the diet) alone or in association with VitE (170 mg/kg) compared with basal diet containing only low levels of VitE (30 mg/kg) increased (by 15.2% on average) broilers’ WG, whereas no significant differences were reported between OP and VitE groups [23]. When broilers were supplemented with a high dose of OP (0.5% of the diet) for 42 days, the overall WG was increased (by 15.9%) compared with the control group, which only contained VitE and VitC at low doses (30 and 10 mg/kg, respectively) [44].

In many cases, the supplementation with different PFA revealed the same effect as the addition of synthetic vitamins. For example, in turkeys, the same BW and WG were obtained by the dietary inclusion of OO (0.010%, 0.015%, and 0.020% of the diet) or a low dose of VitE (100 mg/kg of the diet) compared with greater doses of VitE (200 mg/kg) during a 28 day trial [21]. In a 38 day feeding trial, OO supplemented broilers (0.01% and 0.02% of the diet) did not change their BW compared with those fed a basal (30 mg/kg of VitE) or a VitE rich (200 mg/kg) diet [45]. Similarly, in broilers fed with two different diets (crude soybean oil diet or acidulated soybean oil soapstock), the supplementation with OO (0.01% of the diet) did not significantly affect WG in comparison with two inclusion levels of VitE (10 and 100 mg/kg) in the diet [22]. However, when different doses of OO and RO were included in broilers diet, BW was negatively affected [24]. The lower inclusion levels of RO in broilers diet for 21 days reduced (by 5.1%) their BW compared with those consuming VitE at a basal (only 50 mg/kg of VitE) or at the highest level (200 mg/kg of VitE). The dietary inclusion of OO at the lowest dose alone or in combination with RO (at 0.075% of the diet each) reduced broilers’ BW (by 5.0% average). A reduction (by 5.6% on average) of BW was found in broilers consuming simultaneously OO and RO in two different combinations (0.075% and 0.15% of the diet) at the end of the experiment (42 days). Similarly, the highest dietary inclusion levels of RO and OO compared with the basal diets reduced (by 4.1% on average) the broilers’ WG up to 21 days of age. The highest inclusion levels of both RO and OO as well as their combination with the intermediate doses resulted in a reduction (by 3% on average) of their WG. Lower values of WG were also observed with the highest inclusion level of OO as well as with intermediate and higher doses of its combination with the RO (by 6.0% on average) [24]. In another trial, feeding broilers with a mixture of RO, OO, and FVO (all at the concentration of 0.04% of the diet) for 42 days improved FBW and WG (by 7.65% on average) compared with the basal diet (receiving only 50 mg/kg of diet of VitE) but not compared with VitE (200 mg/kg) enriched diet [25].

The dietary addition of TO (0.01% of the diet) compared with a control diet (VitE deficient) for 42 days led to detrimental effects on broilers’ BW and WG (by 0.58% and 0.6%, respectively). Additionally, the BW was higher (by 2.8% on average) in TO (0.01% or 0.02% of the diet) compared with VitE (100 mg/kg) fed broilers but lower (by 0.92%) than those supplemented with the highest dose of VitE (200 mg/kg). The results of WG were consistent with those of BW [27]. Similar findings were obtained for WG and final body weight (FBW) in broilers and ducks supplemented with different natural antioxidants (at 0.2% of the diet). In particular, tomato skin, orange peel, and green tea leaves were tested in broilers, while RL and orange were evaluated in ducks’ diets in comparison with VitE (200 mg/kg) [46]. In broilers, the dietary inclusion of RL (2.5% of the diet) did not change average WG, daily water expenditure, and mortality compared with VitE administration (200 mg/kg) or with the addition of other antioxidant compounds (rosehip, chokeberry pomace, and nettle) [17]. Additionally, the dietary supplementation with tomato pomace (30%) as a source of VitE did not change WG in chicks during a 21 day trial [47]. The dietary supplementation with a commercial blend of essential oils (0.05% and 0.1% of the diet) compared with VitE (200 mg/kg) for 42 days did not affect FBW of broilers [35]. Similar results were obtained for BW in CE (0.056% of the diet), TE (0.056% of the diet), SE (0.056% of the diet), MX (0.02% of the diet), synthetic antioxidants (0.00486% of the diet), and β-apo-8-carotenoic acid ethylester (0.004% of the diet) compared with VitE (150 mg/kg) fed broilers [19]. Feeding SE (2.5% of the diet) in laying hens did not affect significantly WG and FBW compared with negative control or with VitE treatment (30 mg/kg) [20].

The dietary supplementation with OLE for 14 days was not able to mitigate the detrimental effect of heat stress on WG [26]. The addition of the commercial product Proviox^®^ (PP; Provimi, France; 0.02% of the diet) alone or simultaneously with VitE (PPE; 100 mg/kg of VitE) for 38 days increased (by 18.1%) final BW in HS broilers. Feeding PP also led to an improved (by 13.8% on average) WG compared with both thermoneutral and HS control groups, whereas PPE fed animals showed higher (by 16.8%) WG only compared with the HS group [37]. In a similar study on broilers, feeding PP did not mitigate the detrimental effects of Ochratoxin A contaminated grain [38]. In fact, at day 21 of the trial, BW was lower (by 26.3% on average) in PPE and PP groups compared with those consuming a non-contaminated diet. Similar results were found also after 35 days, when BW values were reduced (by an average of 11.3%). The WG was also lower (by an average of 16.7%) in PPE and PP groups. In another similar study, the same dietary treatments did not affect BW and WG in broilers fed with low quality oil, and no significant differences were observed between PP and VitE fed animals [39].

Concerning growth performances, positive effects or similar results to the positive control (VitE or VitC administration) were obtained using oregano plant both at low and high doses as well as oregano aqueous extract and thyme oil. On the other hand, oregano oil at high doses (>0.06% of the diet) negatively affected growth. It seems that the form (plant vs. extract or oil) of PFA has different effects on growth parameters (e.g., oregano plant vs. oregano oil). Moreover, the highest dietary inclusion doses of PFA (in every form except as a whole plant) generally depressed WG and FBW (e.g., oregano oil, grape pomace, grape seed extract, fermented and unfermented grape skin). Some PFAs (e.g., grape seed extract, *Forsythia suspensa* extract, and a generic commercial polyphenol product) were able to face the impact of heat stress on poultry growth.

Regarding the potential positive effects of PFA on FI and WG, some modes of action were proposed: (i) enhancement of flavor and palatability; (ii) improvement of feed antioxidant status; (iii) decrease of microbial colonization of feed; (iv) and increased appetite stimulation [1,2,40]. Even if some studies reported beneficial effects on FI and WG in broilers [41], other studies reported detrimental effects, probably due to the high inclusion levels of PFA in the diets [12].

#### 3.1.3. Feed Gain Ratio or Feed Conversion Ratio

The GP (from 0.05% to 10% doses) compared with VitE (200 mg/kg) inclusion in broilers’ diet did not impair feed:gain ratio (F:G) after 21 [15] or 42 days [34]. The F:G ratio of broilers and ducks was not affected by the dietary addition of CE, TE, SE, MX [19], RL [17], OAE [42,43], OO [22,45], OO, and RO alone or in different combinations [24], nor were TP [47] and RP [28] affected in comparison with VitE (from 150 to 200 mg/kg). Moreover, in broilers and ducks, the dietary inclusion of different natural antioxidants [46], OLE under HS [26], hesperidin [48], and polyphenols [37,38,39] compared with VitE (150–200 mg/kg) did not affect the F:G ratio. Similarly, the dietary supplementation with RP (0.5% and 1% of the diet) [29,30], OP (0.5% of the diet), or SP (2% of the diet) [29] in comparison with VitE (200 mg/kg of the diet) did not modify the F:G ratio in laying hens. The same results were reported when RL and RO were added in broilers’ diets for 42 days, even though a reduction (by 9%) on the F:G ratio of the RO compared with VitE fed animals (50 and 20 mg/kg) was observed after 21 days [18]. Other studies reported significant effects of PFA on F:G ratio but only in comparison with VitE deficient diets or with diets without VitE. A reduction (by 8.1% on average) of F:G ratio was found when OP alone or in combination with VitE (OP + VitE) or VitC (OP + VitC) was added in broilers’ diet for 14 days [44]. Greater beneficial effects were reported in FSE fed broilers, which decreased (by 34.47%) the F:G ratio [36]. A significant decrease (by an average of 3.8%) of F:G ratio in RL fed broilers compared with those consuming VitE at basal or supplemental levels was found in the initial two weeks of trial [17]. Feeding broilers with TO (0.01% and 0.02% of the diet) increased F:G ratio compared with those consuming no VitE (by 1.4%), VitE at 100 mg/kg (by 4.1%), or VitE at 200 mg/kg (by 2.6%) [27]. Conversely, the administration of a mixture of OO, RO, and FVO (0.04% of the diet each) compared with a basal and VitE rich diet reduced (by 8.1% on average) broilers’ F:G ratio [25].

In addition, a decrease (by 5.9%) of F:G ratio in broilers fed with GP (0.6% of the diet) compared with VitE (200 mg/kg) was observed [14]. Broilers under HS supplemented with FSE also reduced (by 4.4%) their F:G ratio in comparison with those consuming only the basal but not the VitC enriched diet [36]. A decrease (by 12.9%) in F:G ratio was also observed in GTE (0.5% of the diet) fed laying hens [33], but this value was similar to that obtained with the VitE administration (200 mg/kg). Moreover, broilers supplemented with GSE (0.03% of the diet) exhibited greater values (14.8–47%) of the European production efficiency factor (EPEF) than basal and VitC fed ones at 29 and 42 days of trial [40]. The EPEF was calculated as the ratio between the product of viability (%) and the BW and the product of age and the feed conversion rate [49]. Conversely, the dietary supplementation of broilers with FGS and UGS (3% and 6% of the diet each) negatively affected the F:G ratio [16]. Indeed, higher F:G ratio in UGS compared with basal and VitE fed broilers was found (by about 12% and 10%, respectively); moreover, the F:G ratio was also higher in relation to unsupplemented animals (by 13.5%) and to VitE ones (by 11.9%) in comparison with those consuming the diet with the highest inclusion level of FGS. Giannenas et al. [23] reported beneficial effects of feeding OP (0.5% of the diet) alone or mixed with VitE (170 mg/kg) for 42 days on F:G ratio. In fact, the supplemented compared with the unsupplemented broilers reduced (by 5.6%) their F:G ratio without significant differences in the VitE fed ones.

Most of the PFAs tested had no effect on F:G ratio in broilers, ducks, and laying hens. Among the studies, OP alone or in combination with VitE or VitC consistently improved feed use efficiency in broilers. A similar effect was found for *Forsythia suspensa* (also under heat stress), rosemary leaves, and a mixture of oregano oil, rosemary oil, fennel volatile oil, and grape pomace in broilers as well as for the green tea extract in laying hens. On the other side, thyme oil, and fermented and unfermented grape skin increased F:G ratio in broilers with detrimental effect on efficiency. It should be highlighted here that the lack of effect on F:G ratio can also be associated with a reduced feed intake and a depressed growth performance occurring simultaneously, which can be considered a negative effect at farm level.

#### 3.1.4. Nutrients Digestibility

The PFAs exert different biological activities that are related to intestinal tract functions [12]. In fact, a lower nutrient digestibility could be related to the dietary content of polyphenols, which may affect both protein and fat digestibility.

The GP (1.5% of the diet) compared with VitE inclusion in broilers diet did not modify apparent ileal fat digestibility (AIDF) [14]. Nevertheless, the dietary supplementation with GP (3% and 6% of the diet) compared with VitE reduced (by 3.9% on average) the AIDF, whereas the apparent ileal protein digestibility (AIDP) was not affected [14]. The lower AIDF might have been related to the increase of both hydrolysable polyphenols (HP) and condensed tannins (CT) intake, dependent on the use of GP. In fact, broilers fed with the highest inclusion level of GP compared to broilers receiving the lowest level had obviously higher HP (by 18.35%) and CT (by 289.4%) total intake. Moreover, the GP addition at high levels (3% and 6% of the diet) increased HP total intake (by 8.7% and 23.3%, respectively) compared with basal and VitE rich diets [14]. Ileal digestibility and fecal digestibility of HP (IDHP and FDHP, respectively) were reduced by increasing GP inclusion in broilers diet. The IDHP was lower (by 16.0% on average) compared with both control and VitE groups, whereas FDHP was lower (by 16.8%) compared with control and VitE treated animals [14]. The same findings were observed for the AIDP (by a mean value of 6.62%) in the UGS (6% of the diet) fed animals. Moreover, UGS dietary addition (3% and 6% of the diet) reduced ileal total extractable polyphenols (TEP) compared with the basal (by a mean proportion of 18.4%) and the VitE (by an average amount of 22.3%) fed diet [16].

On the other hand, FGS did not significantly affect the above parameters. Broilers supplemented with FSE (0.1% of the diet) for 42 days exhibited greater apparent digestibility of crude protein (on average by 3.3%) and calcium (on average by 5.5%) compared with those consuming basal and VitC diets [36]. The RL (2.5% of the diet) compared with rosehip administration for 5 weeks reduced (by 9.1%) the nitrogen metabolizability of broilers diet and the metabolizable energy (by 6.4% on average) compared with the control, the VitE, and the nettle groups [17].

A greater apparent digestibility of energy (by 3.9%) and phosphorus (by 12.2%) in FSE fed animals was found [36]. The dietary supplementation with OLE (0.4% of the diet) in HS broilers for 14 days lowered apparent digestibility of energy (by 8.5%) compared with VitE fed animals but not in comparison with those consuming the basal diet where this parameter was increased (by 2.7%). The OLE fed animals (0.4% of the diet) showed a reduction in apparent digestibility of crude protein (by 14.5%), ash (by 22.7%), and phosphorous (by 15.7%) [26].

From the studies reported above, it appears that the only positive effects on protein and mineral digestibility were related to the use of *Forsythia suspensa* extract, while negative effects on this parameter were exerted by grape pomace, unfermented grape skin, rosemary leaves, and olive leaves extract. As already highlighted, the lower digestibility might be related to the increase of both hydrolysable polyphenols and/or condensed tannins intake associated with the use of PFA.

### 3.2. Effects on Metabolic, Hematological Parameters, and Antioxidant Status (Table S2 in Supplementary Materials)

#### 3.2.1. Serum Biochemical Parameters

Serum lipid profile (cholesterol -CHOL triglyceride -TG high-density lipoproteins (HDL)) was evaluated in laying hens when clove bud powder (CBP; 0.2% and 0.4% of the diet) or VitE (200 mg/kg) were incorporated in diets with high (HFA) or low (LFA) n-6 to n-3 fatty acids ratio (n-6:n-3) [50]. The addition of CBP (0.2% of the diet) or VitE in an HFA diet decreased CHOL to the same extent (by 17.1% and 18.5%, respectively). However, the highest inclusion level of CBP compared with VitE and basal diets was more effective in decreasing (by an average of 27.90%) CHOL. By contrast, VitE, when incorporated in LFA diet, was more effective in decreasing CHOL (by 11.7%) compared with the unsupplemented dietary treatment. However, CBP addition in both HFA and LFA diets was more effective than VitE and unsupplemented treatments in reducing CHOL (by 16.0% and 25.8%, respectively). A linear decrease in serum CHOL by increasing the inclusion levels of CBP in laying hens’ diet was observed. This could be related to the hypocholesterolemic property that some plant molecules—usually found in essential oils—may exert [51,52], mainly through the reduction of 3-hydroxy-3-methylglutaryl-CoA reductase (HMGCoA), which is the rate-limiting enzyme involved in CHOL synthesis [53]. Moreover, CB essential oil contains several bioactive compounds, among which is eugenol, which was also demonstrated to possess antioxidant properties [54].

In broilers, the lower dose of GP (5%, 7.5%, and 10% of the diet) inclusion in an unspecified acid profile diet was not effective in decreasing total CHOL. The same results were observed using VitE (200 mg/kg). By contrast, GP (10.0% of the diet) compared with VitE or a basal diet increased HDL-C (by 50.1% on average). However, all the GP compared with control fed animals showed a significant decrease (by 31.0% on average) for LDL-C, whereas only the highest inclusion level of GP showed lower (by 28.6%) LDL-C compared with VitE [34]. As the authors suggested, the mechanism by which GP by-product could affect lipid metabolism is not well established yet. Nevertheless, the HDL-C increase in this study could be associated with the capacity of phenolic molecules contained in GP by-products to scavenge free radicals and consequently to reduce oxidized lipid products [5].

In contrast to these findings, the dietary inclusion of another vinery by-product in broilers, namely grape seed extract (GSE; 0.015–0.045% of the diet), did not have any significant effect on CHOL, HDL-C, or LDL-C parameters in comparison with basal and VitC (300 mg/kg) diets under thermoneutral conditions [40]. By contrast, under HS conditions, VitC and GSE (0.015% and 0.030% of the diet) fed animals, compared with the unsupplemented ones, showed lower CHOL (by an average of 8.4%) level. The lower level of GSE was not effective in decreasing CHOL concentration, whereas LDL proteins were lowered (by 27%) only with the highest inclusion amount in comparison with the basal diet. Similarly, the highest inclusion level of GSE compared with the basal diet resulted in lowering (by 10.7%) the very low density lipoproteins (VLDL). Based on these results, it seems that HS plays a key role in lipid metabolism of broilers, affecting also GSE impact, since stressors affect CHOL level in plasma of poultry by the stimulation of the adrenal grand, which induces lipolysis.

Laying hens supplemented with green tea powder (GTP; 0.5% of the diet), GTE (1.5% of the diet), marigold powder (MP, 0.5% of the diet), or marigold extract (ME, 1.5% of the diet) presented lower serum LDL and CHOL levels in comparison with the unsupplemented animals, which expressed an effect similar to those consuming the VitE (200 mg/kg) diet. In addition, reductions (by 7.3% and 18.2%, respectively) of serum TG and HDL/CHOL were only observed for the GTE supplementation when compared with the basal diet [33]. Consistently, green tea (*Camelia siniensis*) polyphenols, such as catechins, were demonstrated to alter lipid metabolism in broilers’ livers [55].

The CBP or the VitE inclusion in HFA compared with basal diet decreased (by 19.4% on average) the TG levels in laying hens. Increasing CBP levels of the diet led to a linear decrease of TG content compared with the basal (by 39.6%) and the VitE (by 25.9%) treatments [50]. In LFA diets, the addition of CBP compared with basal and VitE diets decreased (by averages of 13.3% and of 28.8%, respectively) TG content [50].

Feeding GP to broilers led to a reduction of TG (by an average of 39.4%) in comparison with the control group, without significant differences between GP and VitE groups [34]. In contrast to these findings, broilers reared under thermoneutral conditions and fed with GSE in comparison with VitC did not show any significant effect on TG levels [40]. Nevertheless, in the same study, the highest GSE supplementation level led to a significant decrease of TG concentration (by 10.8%) compared with the basal diet. However, no significant differences between VitC and GSE groups or between VitC and control groups were found. In the same study, glucose was lower in GSE compared with the basal (by an average of 12.0%) and the VitC (by about 8.6%) fed animals after 28 days of trial, whereas no differences between VitC and control groups were observed. At day 42, lower values in GSE than control (on average 15.6%) and VitC groups (mean of 11.7%) were reported [40]. Conversely, TG and CHOL were not significantly influenced by feeding anise seed (AS; 1% and 2% of the diet) in Japanese quails in comparison with VitE (600 mg/kg) [32]. Only feeding AS (at 1% of the diet) tended to decrease (by about 23.4%) TG concentration compared with control and VitE [32].

Broilers reared under HS and fed a diet with olive leaves added (0.2% and 0.4% of the diet) or supplemented with VitE (250 mg/kg) had a lower CHOL than control, with the lowest value in OL with a lower dose than the control group (by 15.5%) [26]. The authors also reported lower and similar TG values in OL and VitE groups in comparison with control animals (on average reduced by 34%). Uric acid was also lower in OL with a higher dose than in OL with a lower dose and in VitE groups (by 28.9% on average) as well as in the control (by 0.48%).

Significant changes of lipid metabolism parameters were observed when TO was supplemented in broilers [27]. More in detail, broilers supplemented with TO (0.01% and 0.02% of the diet) increased total CHOL (by 7.1% and 10.3%) in comparison with groups supplemented with VitE (100 and 200 mg/kg, respectively). Furthermore, HDL-C was higher (by 8.9%) in the case of TO inclusion at the lower level and lower (by an average of 4.2%) when TO was supplemented at the higher level in comparison with the VitE groups. Similarly, LDL-C was higher (15.7% by average) in TO treated animals than in VitE groups [27]. Finally, the TO supplementation increased all the mentioned lipid metabolism markers when compared to the control group. This study shows clearly the complete lack of the expected hypocholesterolemic effect of thyme oil.

Several PFAs (e.g., grape pomace, grapeseed extract, clove bud powder, green tea extract and powder, as well as marigold extract) had a general positive impact on lipid metabolism by decreasing cholesterol and increasing the HDL levels in a similar way or in greater extent in comparison with negative and positive controls. Among the tested PFAs, thyme oil was the only one that had negative effects on the serum biochemical parameters related to lipid metabolism.

#### 3.2.2. Serum Enzyme Activities

Other parameters investigated were serum enzyme activities, such as aspartate aminotransferase (AST), alanine aminotransferase (ALT), alkaline phosphatase (ALP), and lactate dehydrogenase (LDH). In general, supplementation with PFA is expected to exert a hepatoprotective effect, reducing liver damage markers as it was demonstrated in laying hens [56]. Particularly, in this study, the CBP addition in either high or low n-6:n-3 diets (HRD and LRD, respectively) reduced ALT, AST, and ALP activities. The same effect on these enzymes was obtained by decreasing n-6:n-3 of the diet [50]. In HRD fed laying hens, the CBP inclusion (0.2% and 0.4% of the diet) reduced AST (by an average of 54.8%) and ALT (by 20.8%) activities. In addition, CBP was more effective in decreasing AST and ALT activities than VitE, which was, however, effective in both dietary conditions. Hens consuming the highest level of CBP showed a more intense reduction of AST and ALT serum activities when compared to those fed high levels of VitE in both HRD and LRD. However, VitE was also reported to be able to reduce serum AST and ALT activities [50].

On the other hand, broilers exposed to HS conditions (34 °C) for 41 days and fed with GP (10% of the diet) increased (by 63.5%) their AST activity in plasma compared to those fed with control and VitE (200 mg/kg) diets [34]. As suggested by the authors, the higher AST might have been related to the hepatocellular necrosis potentially inducted from polyphenols. The potential hepatoprotective effect of PFA was also confirmed in another study [26]. The dietary supplementation with OLE (0.4% of the diet) or VitE compared with a basal diet reduced ALT activity (by 18.0% and 25.8%, respectively). The OLE compared with VitE and control diet was also able to reduce (18.1% by average) ALP activity [26]. These positive findings could be partially explained with the preventive action of olive leaves towards free radicals’ generation, hence preserving hepatocytes integrity.

To summarize the above finding, it appears that clove bud powder and olive leaves extract were the main PFAs which decreased serum hepatic enzymes activity. The opposite effect was observed when high levels of grape pomace were included in the diet.

#### 3.2.3. Plasma Oxidative Status

Several studies investigated the effects of PFA on plasma oxidative status markers. Among these, of importance is the superoxide dismutase (SOD), which, along with glutathione peroxidase (GPx), acts as an antioxidant in reactive oxygen species (ROS) balance. Broilers fed with RL (0.57%, 0.86%, and 1.15% of the diet) and RO (0.01%, 0.015%, and 0.02% of the diet) compared with VitE (50 and 200 mg/kg) for 42 days did not show significant differences on SOD activity, whereas the high inclusion levels of RL and RO reduced this parameter [18]. Similarly, no significant effects on broilers’ SOD activity were observed in a trial comparing the dietary inclusion of OL with VitE [26]. The dietary supplementation with GP (7.5% of the diet) in broilers reared under thermoneutral conditions increased plasma SOD activity compared with control and VitE (by an average proportion of 50.8%) groups. The dietary supplementation with GP (5% of the diet) compared with basal and VitE diets led to a similar increase of SOD activity but lower in extent. Meanwhile, the dietary inclusion of GP at higher level (10% of the diet) did not improve SOD activity compared with basal or VitE (200 mg/kg) diets [34].

Broilers showed higher GPx activity when fed with GP (5% of the diet) in comparison with those consuming a basal (by 46.3%) diet. Higher doses of GP (7.5% of the diet) compared with basal and VitE diets increased GPx activity (by a mean value of 46.4%), whereas VitE compared with the basal diet did not improve this parameter [34]. The dietary supplementation with OLE compared with a basal diet increased GPx (by 24.0%) but not when compared with a VitE diet [26].

Another parameter investigated to assess the antioxidant capacity of PFA was malonildialdehyde (MDA), an endogenous marker of lipid oxidation, whose increase is induced by a decreased antioxidant protection capacity of the organism. Several studies reported favorable effects of supplementing poultry diet with PFA on different markers related with the antioxidant status. For instance, the dietary inclusion of OL (0.4% of the diet) compared with VitE and a basal diet reduced MDA values (by 22.5%) in plasma. A reduction of the ferric reducing activity power (FRAP by 19.1%) in broilers fed with OL (0.4% of the diet) compared with VitE was found. However, FRAP was higher (by 26.5%) in broilers fed the basal diet [26]. The GP fed animals (7.5% of the diet) compared with those consuming basal and VitE diets reduced their MDA plasma content (by 25.2%) [34]. A high n-6:n-3 diet (HDR) combined with CBP (0.4% of the diet) lowered MDA content (by a mean proportion of 28.3%) in laying hens compared with basal and VitE diets. The same CBP treatment induced a decrease of hepatic MDA content (by 29.7%) compared with control, low levels of CBP (0.2% of the diet), and VitE diets. When the same animals were fed with a low n-6:n-3 diet (LRD) supplemented with CBP at the high level (0.4% of the diet) compared with those consuming basal, CBP at low level (0.2% of the diet), and VitE, treatments decreased their MDA plasma content (by 24.2% on average). The same group had also the lowest hepatic MDA content (by 13.8%) compared with control, CBP (0.2% of the diet), and VitE. The lower level of CBP dietary inclusion was as effective as VitE in reducing this parameter [50]. The dietary supplementation with CBP at both levels also significantly increased total antioxidant capacity (TAC) independently from n-6:n-3 ratio in the diets (HRD and LRD diets). The HRD fed animals, when supplemented with CBP at both levels, showed higher TAC than those consuming the basal (by 41.5% on average at the two doses) and the VitE (by 17.5% on average) diets. Moreover, the LRD fed animals, when supplemented with the high level of CBP only, showed higher TAC than those fed basal and VitE diets (by 51.0% on average). These results showed that the highest inclusion levels of CBP were more effective than VitE in improving TAC values. Furthermore, CBD supplementation in the HRD diet increased VitE content in blood in comparison with the basal diet (by an average of 79.5% at the two doses). Nevertheless, only the highest inclusion level of CBP in LRD diet increased VitE level (by 103.0%) [50].

By contrast, when a sweet chestnut wood (SCW) extract was fed to broilers in comparison with high polyunsaturated fatty acids (PUFA; from linseed oil) content diets and two levels of VitE (85 and 200 mg/kg), no significant effects were reported on plasma and liver MDA content, total antioxidant status (TAS), lipid soluble antioxidant capacity (ACL), or SOD and GPx activities [57]. The SCW feeding reduced (by 42.4%) the DNA damage compared only to the linseed oil fed group [57]. In broilers fed with natural tocopherols, rosemary, grape seed, green tea, and tomato alone or in different combinations (0.01% and 0.02% of the diet) among them for 42 days, no significant effects on plasma FRAP, thiobarbituric acid reactive substances (TBARS), or SOD activity were reported. Conversely, some effects were observed on GPx activities [58].

The TAS was not affected by polyphenols (PP) supplementation in broilers’ diet in three similar studies [37,38,39]. However, SOD activity was reduced in PP (by 18.3%) and PPE (PP with VitE; by 21.8%) fed animals compared with those not exposed to heat stress (thermoneutral). The GPx activity was increased (by 49.1%) in the PPE group compared to those challenged with heat stress [37] as well as in PPE (by 20.5%) and in PP (by 11.9%) groups in comparison with those consuming a low quality oil diet [39]. In this last study, GPx activity was higher in PPE (by 15.0%) and in PP (by 14.3%) fed animals. In another similar study in which all diets contained grain contaminated with ochratoxin A, TAS was greater in PPE (by 23.5%) and PP (by 21.6%) groups than in the positive control group (only grain contaminated) [38]. However, SOD activity was lower (by 18.4%) in PP compared with the control group, which consumed the uncontaminated grain, but was higher (by 10.6%) in PPE than the VitE (100 mg/kg) group and lower in PP (by 12.8%) than the high dose VitE group (200 mg/kg). In the same study, GPx activity was higher in PPE (by 17.6%) and in PP (by 34.6%) than the contaminated grain fed broilers without showing any significant difference with VitE. Among different parameters investigated (VitE, total tocopherols, VitC, retinol) in blood and in liver, hepatic TBARS values were significantly affected. Indeed, they were higher (by 32.0% on average) in PPE and in PP in comparison with the thermoneutral group [37]. Conversely, the supplementation with PPE decreased hepatic TBARS values compared with broilers fed with contaminated grain, demonstrating a synergistic effect of PP and VitE in mitigating the detrimental effects of nutritional stress [38].

Dietary supplementation of chickens with GP (3% of the diet) compared with basal and VitE diets resulted in a higher total intake of extractable polyphenols (by 50.6%) [15]. Digestibility of extractable polyphenols was also higher (on average 117.2%) in GP than in basal and VitE fed animals [15]. Total antioxidant capacity (measured by ABTS assay) of chicken excreta increased (by 19.9%) when fed with GP compared with basal diet. A similar increase (by 18.8%) was found when the TAC was determined by FRAP assay. No difference was observed between VitE and GP treatments in ameliorating this marker [15]. In male broiler chicks, TAC was also affected when GP was included in animals’ diets at different levels (1.5%, 3%, and 6% of the diet) [14]. In fact, TAC of diet, ileal content, and excreta were higher (by average proportions of 345.4%, 102.7%, and 41.6%, respectively) in GP than in unsupplemented broilers. Meanwhile, the same parameter was determined to be higher (by a mean of 125.6%) in the diet and the ileum (by an average of 68.2%) in GP than in the VitE (200 mg/kg) fed animals. Furthermore, GP supplementation did not affect the serum TAC (measured by ABTS assay). On the other hand, the total antioxidant capacity (measured by the FRAP assay) differed between GP and basal diet fed animals [14].

In laying hens, the dietary supplementation with Roselle (*Hibiscus sabdariffa* L.) calyx (crude extracts powder at the 2.4% concentration administered at the dose of 1% or 2% of the diet) did not change the TBARS values in plasma in the first 4 weeks of an 8 week feeding trial among the different inclusion levels in the diet or in comparison with the VitE supplementation (250 mg/kg). Nevertheless, an overall tendency in decreasing this parameter was determined by Roselle calyx in yolk [31].

In broilers fed a linseed oil rich diet (high PUFA), the addition of SCW (0.3% of the diet) reduced the lymphocyte DNA damage (by 42.0%) [57]. Lower relative transcripts levels (by 594.3% on average) of HSP70 gene (expression of which usually increases in heat stress) in the heart tissue of GSE fed broilers were found when animals were reared under heat stress. A reduction (by 199.9%) in the expression of HSP70 gene in the liver of GSE fed broilers exposed to heat stress was also found [40].

The effects of PFA on selected immunological parameters in comparison with synthetic vitamins were also investigated in poultry. More specifically, different grape polyphenols (GPP) to VitE ratios (C, 0:100; LGPP, 25:75; MGPP, 50:50; HGPP, 75:25) in broiler diets were tested. Broilers supplemented with the highest GPP:VitE ratio (HGPP) compared with those receiving only VitE (100 mg/kg) improved their antibody titers (by a mean value of 59.5%) against Newcastle disease and infectious bursal disease (IBD) [59]. However, antibody titers against IBD were also higher in HGPP compared with LGPP and MGPP groups (by an average proportion of 389.0%) [59]. In contrast to these results, dietary supplementation with rosemary powder (RPO; 0%, 0.5%, and 1% of the diet) or VitE for 42 days did not affect broilers’ antibody titers against viruses and sheep red blood cells (SRBC) [60].

In most of the cases, the dietary addition of PFS was able to reduce the MDA content of plasma and tissues. Based on the type of PFA, this decrease was accompanied by changes in antioxidant enzymes activities and total antioxidant capacity. Grape pomace and grape polyphenols positively affected poultry immunity due to an increase in antibody titers.

### 3.3. Effects on Macro- and Microscopic Anatomical Features and on Intestinal Microbiome Profile (Table S3 in Supplementary Materials)

Several studies suggested an immunostimulant activity exerted by polyphenols on different signal transduction pathways, which are responsible for a wide range of cell functions related, for instance, to immune organs growth and proliferation [61].

The broiler spleen and bursa of Fabricius were not affected when their diet was supplemented with different levels of GP (5%, 7.5%, and 10% of the diet) or with VitE (200 mg/kg) [34]. Similarly, lower GP supplementation levels (1.5% to 6% of the diet) did not affect relative spleen weight in broilers; only those that were supplemented with low GP levels (1.5% of the diet) compared with VitE showed improvement in this morphological parameter (by 20.0%) [14]. Similar findings concerning weights of thymus, spleen, and bursa of Fabricius in RPO (0.5% and 1% of the diet) and VitE (100 and 200 mg/kg) fed broilers were found [60].

Furthermore, some studies also tested PFA effects on small intestine and gut gross morphometry, which have relevance for digestion capability. Broilers supplemented with GP (1.5% of the diet) compared with VitE improved relative length of duodenum and ceca (by about 9.0%) [14]. On the other hand, RPO addition (0.5–1% of the diet) in broilers’ diet negatively affected jejunum length and weight, colon length and weight, and right cecum weight, but no significant differences were reported concerning these anatomical traits compared with those consuming two different levels of VitE (100 and 200 mg/kg) [28]. The dietary supplementation with GP (5%, 7.5%, and 10% of the diet) for 42 days affected broilers’ mucosa status and microscopic structure [34]. The GP supplemented compared with the unsupplemented broilers showed a significant reduction (by 35.2%) of villus height (VH) in jejunum, while those consuming the highest levels of GP had also shorter villus height (by 35.4%) compared with the VitE fed ones. Moreover, broilers supplemented with GP (7.5% and 10% of the diet) in comparison with the unsupplemented ones had significantly lower VH/crypt ratios above all in jejunum (with a mean reduction of 35.2%), whereas only those consuming the highest GP level compared with those receiving VitE showed a lower value (by 35.4%) [34]. This study confirmed the potential effect of polyphenols on intestinal digestive and absorption functions.

In laying hens, when clove bud powder (CBP) was included at two different levels (2% and 4% of the diet) in two diets with different n-6:n-3 in comparison with VitE (200 mg/kg), it did not affect liver relative weight [50]. Nevertheless, when CBP (0.4% of the diet) was included in a HRD diet, it increased the hepatic tissue integrity score (HTI; by an average value of 47.8%) in laying hens. Moreover, fat vacuole numbers score (FVN) was reduced by consuming the CBP compared to the basal diet (on average 36.5%), but the VitE diet resulted in lower FVN value than the CBP treatments. The CBP inclusion (0.2% of the diet) in LRD diet increased the HTI score (by 46.37% on average), whereas the FVN score was lower (25.9–51.8%) in all the CBP fed animals in comparison with those consuming VitE (by 35.0%) [50]. These results suggest that reducing n-6:n-3 led to a detrimental effect on HTI; all the experimental groups exhibited higher values of HTI when fed LRD diets. However, both CBP and VitE exerted hypocholesterolemic and hypolipidemic effects, which were confirmed by lower FVN and greater HTI scores.

Conversely, grape polyphenols addition (0.0025%, 0.0050%, and 0.0075% of the diet) in broilers’ diet did not cause any significant effects on histopathological features of kidneys and liver [59].

Among the potentially beneficial effects of PFA on avian health and performances, some studies investigated their effectiveness in controlling intestinal microflora and mucosa integrity [62]. Dietary supplementation with oregano aqueous extract (OAE; 0.02% of the diet) compared with VitE and unsupplemented diets positively affected gut secretion glyconjugates [42] by the goblet cells, which cover the gastrointestinal mucosal layer and have an important role in protecting the intestinal mucosa [63].

The PFA effectiveness in controlling pathogens is of great interest in livestock species, including poultry, especially in some critical phases of animals’ lifespan [10]. Coliforms bacteria count was lower in OAE than in VitE and control groups (by an average proportion of 20.8%) [42]. Similar results were obtained in the following interval. OAE compared with VitE dietary inclusion reduced the *Escherichia coli* count in broilers caecum at 21 (by 35.0%) and 42 (by 9.0%) days, respectively. Coliform species are of great interest, because some of them are pathogenic organisms, sometimes representing also a zoonotic threat [64,65]. The dietary inclusion of OAE in comparison with VitE and unsupplemented diets for 3 weeks lowered (by 32.5%) ileal count of total anaerobia bacteria. At the following interval, this dietary treatment induced higher bacterial counts [42]. Additionally, in broilers’ ileum, no differences were found among the treatments groups concerning *Staphylococcus* spp., while lactic acid bacteria (LAB) increased in the OAE group throughout the time interval of 21 days. However, the count of these beneficial bacteria did not differ significantly among the three groups. Nevertheless, enterococci, lactobacilli, and staphylococci populations in caecum at both sampling times were not significantly affected by the different treatments [42]. Consistent with the latter work, UGS and FGS supplementation in broilers did not affect significantly LAB, *E*. *coli,* and *Clostridium* spp. populations in the ileal contents of 21 day broilers [16].

These studies show that the dietary inclusion of PFAs may have some effects on macro- and microscopic anatomical features and on intestinal microbiome profiles. However, the results are not consistent, and further research needs to be done in this field.

## 4. Final and General Remarks

The aim of the present work was to summarize the scientific evidences published in peer-reviewed journals concerning PFA use as alternative sources of antioxidants in poultry. The major part of the scientific papers is related to PFA and VitE as the most relevant synthetic antioxidant administered to the animals through the feedstuff, while only a few studies are available regarding VitC. Even if the effect of the PFA on the antioxidant status of the animals represents the main concern in this review, several other traits were investigated since null or positive effects on these relevant physiological functions of poultry are fundamental for their productivity.

In general, both positive and negative effects were found in poultry by different researchers in relationship to the administration of PFAs on several animal functions and conditions, including growth, feed intake, feed utilization performances, blood biochemical parameters, antioxidant status, immunity, gene expression, and gut/tissues development.

Concerning growth performances, positive effects or similar results to the positive control (VitE or VitC administration) were obtained using oregano plant, rosemary volatile oil, *Forsythia suspensa* extract, and a generic polyphenol product as well as rosemary, oregano, and fennel volatile oil. A positive or neutral effect on feed use (F:G ratio) was found for oregano plant, green tea, and marigold extract or powder, which reduced feed intake, as well as for rosemary, oregano, and fennel volatile oil, which reduced F:G ratio by increasing growth. Positive effects on energy, protein, and mineral digestibility were found also in relationship to the use of *Forsythia suspensa* extract. However, negative effects on digestibility were exerted by grape pomace and fermented and unfermented grape skin.

The main biochemical traits investigated were those related to the lipid metabolism. A generalized positive effect was found in relationship to the capacity of several PFAs to increase HDL in a similar way or to a higher extent in comparison with negative and positive controls. This effect was shared by grape pomace, grapeseed extract, clove bud powder, green tea extract and powder, and marigold extract. In addition to this, grapeseed extract, clove bud powder, green tea extract and powder, marigold extract, anise seed, and thyme oil were demonstrated to decrease cholesterol in blood.

Concerning the antioxidant status of poultry, among the parameters considered, a significant reduction of blood plasma MDA level was induced by grape pomace, clove bud powder, and olive leaf extract. Conversely, an increase of MDA content was induced by sweet chestnut in comparison with the control group. Some effect was observed also on antioxidant enzymes activity, such as GPx, which was increased by grape pomace, rosemary, and natural tocopherol. On the other side, GPx activity was improved by olive leaf extract, whose effect was also positive on antioxidant capacity measured as FRAP assay.

Poultry immunity was demonstrated to be affected by grape pomace and polyphenols in terms of increased antibody titers in comparison with negative and positive controls, respectively, while no effect was exerted by rosemary powder. A demonstration of the capacity of PFA to modify gene expression was given, for example, by grapeseed extract, whose use increased the expression of HSP in comparison with control groups.

The PFA can affect gut and tissues structure and function, exerting some effects also on microbiota. An example was given again by grape pomace, which was shown to decrease villus height, crypt depth, and mucosal thickness, especially in comparison with negative control, increasing at the same time organs weight and intestine length. Clove bud powder improved tissue integrity and cell physiology, while, in another experiment, grape polyphenols had no effect on lymphoid organs.

The form of PFA can affect the main physiological functions in different ways. oregano decreased growth performances if administered as essential oil but showed a similar effect to VitE, VitC, and their association when included in poultry feed as a whole plant powder. In some cases, the effect seemed to be dose-dependent, such as in the case of grape pomace seed extract, which seemed to exert positive effects on growth performances at low doses but an opposite effect at higher doses with a partial depression of protein digestion and positive effect on HDL levels.

Concerning the context in which each experiment was conducted, the application of environmental or nutritional stressors (e.g., HS, high PUFA diet) was demonstrated to affect the action of PFAs, decreasing, in general, their beneficial effects, probably due to the higher metabolic rate of the organism involving a higher production of free radicals or to the higher susceptibility of the nutrient substrates to oxidative stress.

## 5. Conclusions

The use of PFAs as antioxidants in animal diets was proven to be a viable solution to counteract oxidative stress in several cases, often with a positive impact also on poultry productivity and efficiency. In fact, PFAs appear to have potential to partially or completely substitute antioxidant vitamins in the diet, exerting, at the same time, some side effects on important organic functions of the animals. In some cases, they expressed a synergistic effect with synthetic antioxidants. However, some form- and dose-dependent negative impacts on growth performances and oxidative status were also observed. In any case, the literature on the use of plant feed additives denotes a general variability of the experimental protocols employed to test their activity as antioxidants or their general impact on animal performance and metabolism. A standardization of these protocols is needed.

## Data Availability

The data presented in this study are available on request from the corresponding authors.

## Supplementary Materials

The following are available online at https://www.mdpi.com/article/10.3390/antiox10050659/s1, Table S1: Effects of different plant feed additives on feed utilization and growth parameters in poultry, Table S2: Effects of different plant feed additives on metabolic, hematological, immunological, oxidative stress parameters in poultry, Table S3: Effects of plant feed additives on gastrointestinal anatomic features and microbiota in poultry.
