# Supplementary material for: Plant Feed Additives as Natural Alternatives to the Use of Synthetic Antioxidant Vitamins on Poultry Performances, Health, and Oxidative Status: A Review of the Literature in the Last 20 Years
Source: Antioxidants (Basel). 2021 Apr 23;10(5):659. doi: 10.3390/antiox10050659 (PMC8146777; doi:10.3390/antiox10050659)
Supplement: Supplementary file 1 [file antioxidants-10-00659-s001.zip › Supplementary file_Final_ID1174725_Final.docx.pdf]

Table S1. Effects of different plant feed additives on feed utilization and growth parameters in poultry.

PFA	Dose extract (% of the diet, as FED)	Vitamin of comparison	Class of parameters	Traits evaluated	Comparison to negative control: effect (% of variation, PFA dose)	Comparison to positive control: effect (% of variation, PFA dose)	Period of study (days, d; weeks, wk)	Reference
Grape pomace	5, 7.5, 10	E	Growth performances (GROPerf)	BW, ADG	NS	NS	42 d	Ebrahimzadeh et al. (2018)
			Feed utilization performances (FUPerf)	F:G, ADFI	NS	NS		
Grape pomace	1.5, 3, 6	E	FUPerf	FC	NS	NS	42 d	Brenes et al. (2008)
				F:G	NS	↑5.92% (6%)		
			GROPerf	BW,ADG	NS	NS		
			Nutrient digestibility (ND)	AIDP	NS	NS		
				AIDF	↓2.6% (6%)	↓3.34% (3%) ↓4.5% (6%)		
			AA in diets, ileal content, excreta	TIHP	↑ from 8.7% to 17.8%	↑ from13.6% to 23.2%		
				IDHP	↓ from 12.1% to 25.5%	↓ from 11.6% to 26.1%		
				FDHP	↓ from 6.4% to 29%	↓ from 5.5% to ↓28%		
				TICT	NS	NS		
				IDCT	NS	NS		
				FDCT	NS	NS		
Grape pomace	0.5,1,3	E	GROPerf	WG	NS	NS	21 d	Goni et al. (2007)
			FUPerf	FI, FE	NS	NS		
			ND	AIDP	NS	NS		

				AIDEA	NS	Arg:↓from 0.64% to 0.74%; Leu: from ↓0.54% to 1.72%; Phe: ↓ from 1.5% to 3.5%; Glu: ↓ from 1.8% to 3.96%; Pro: ↓ from 3,89% to 3.75%; Tyr: ↓ from 1.39% to 2.1%; Cys: ↓ from 3.56% to 7.8%		
Grape seed extract	0.015, 0.03,0.045	C	GROPerf	BW at day 28 (pre-heat stress condition)	↑9.3% (0.03%), ↑8.6% (0.015%)	↑5.1% (0.03%)	42 d	Hajati et al. (2015)
				BW at day 42 (under heat stress condition)	↑17.6% (0.03%)	↑9.9% (0.3%)		
			FUPerf	EPEF (1-28 d)	↑14.8% (0.03%) ↑15.9% (0.015%)	NS		
				EPEF (28-42)	↑ 47.1% (0.03%)	↑27.7% (0.03%)		
				EPEF (1-42 d)	↑17.4% (0.045%) ↑34.8% (0.03%) ↑17.5% (0.015%)	↑ 19.4% (0.045%)		
			Livalibility (d 1-42)		NS	NS		
Grape seed extract	2.59, 5.18	E	GROPerf	AFW,WG	Statistical analysis not performed, however very severe growth depression in GSE groups	Statistical analysis not performed, however very severe growth depression in GSE groups	21 d	Lau and King (2003)
Fermented (FGS) , unfermented (UGS) grape skin	3, 6	E	GROPerf	ADG (1-21d)	↓11.14% (6%, FGS) ↓10.62% (6%, UGS)	↓14.68% (6%, FGS) ↓14.18% (6%,UGS)	21 d	Nardoia et al. (2020)
			FUPerf	ADFI (1-21 d)	NS	NS		

				F:G	↑13.53% (6%, FGS) ↑12.03% (3%, UGS) ↑12.78% (6%, UGS)	↑13.53% (6%, FGS) ↑12.03% (3%, UGS) ↑12.78% (6%, UGS)		
			TEP	Ileal	↑12.68% (3%, UGS) ↑22.54% (6%, UGS)	↑8.6% (3%, UGS) ↑18.1% (6%, UGS)		
				Excreta	↑11.44% (3%, UGS) ↑18.77% (6%, UGS)	↑15.15% (3%, UGS) ↑22.73% (6%, UGS)		
			Ileal digestibility of protein and ileal and excreta digestibility of TEP in broiler chickens (21 d)	Ileal protein digestibility	↓6.97% (6%, UGS)	↓6.27% (6%, UGS)		
				Ileal TEP digestibility	↓14.53% (3%, UGS) ↓22.32% (6%, UGS)	↓18.64% (3%, UGS) ↓26.05% (6%, UGS)		
				Excreta TEP digestibility	↓6.75% (3%, UGS) ↓11.04% (6%, UGS)	↓5.88% (3%, UGS) ↓10.25% (6%, UGS)		
				Excreta moisture content	NS	NS		
Tomato pomace	30	E	GROPerf	WG	NS	NS	21 d	King and Zeidler, 2004
			FUPerf	F:G	NS	NS		
Oregano plant	0.5,1, 0.5+ 170 mg/kg of VitE (OR5E), 1+ 170 mg/kg of VitE (OR10E)	E, Flavomicin + lasalocid group (positive control)	GROPerf	WG (42 d)	NC: ↑17.7% (0.5%), ↑12.84% (OR5E); FL: NS	NS	42 d	Giannenas et al., 2005
			FUPerf	FI	NS	NS		

				F:G (35d)	NC: ↓4.7% (0.5%)	NS		
				F:G (42 d)	NC: ↓5.8% (0.5%), ↓5.3% (OR5E)	NS		
Oregano aqueous extract	0.2	E	GROPerf	BW (21 d)	↑8.1%	↑11.46%	42 d	Scocco et al. (2017)
				BW (42 d)	NS	NS		
				F:G	NS	NS		
Oregano oil (turkeys)	0.01,0.02	E	GROPerf, FUPerf	FI, BW, WG,	NS	NS	28 d	Papageorgiou et al., 2003
Oregano essential oil	0.1,0.2	E	GROPerf, FUPerf	BW, F:G	NS	NS	38 d	Botsoglou et al., 2002
Rosemary plant (RP) (laying hens)	0.5,1	E	GROPerf, FUPerf	FI, F:G, FBW	NS	NS	60 d	Florou-Paneri et al., 2006
Rosemary leaves (RL), rosemary volatile oil (RO)	RL:0.57, 0.86,1.15; RO: 0.01, 0.015,0.02	E, 50 mg/kg (E50), 200 mg/kg (E200)	GROPerf	ADG (0-21d)	NA	↑5.2% (RO, average of the 3 inclusion rates)	42 d	Yesilbag et al., 2011
				ADG (21-42d)	NA	↓4.8% (RL)		
				ADG (21-42d)	NA	↓3.2% (RL)		
			FUPerf	FI	NS	NS		
				F:G (0-21 d)	NA	↓9% (RO)		
				F:G (21-42d)	NA	NS		
				F:G (0-42d)	NA	NS		
Rosemary powder	0 0.5, 1	E,100 mg/kg (E100), 200 mg/kg (E200)	GROPerf	WG (1-21d), (22-42d), (1-42d)	NS	NS	42 d	Rostami et al., 2015
			FUPerf	FI (1-21d)	NS	E200:↓4.3% (1%)		
				FI, F:G,	NS	NS		
Mixture of herbal essential oils	0.05,0.1	E	GROPerf	FBW, ADG,	NS	NS	42 d	Botsoglou et al., 2004
			FUPerf	FI, F:G	NS	NS		

Rosemary, oregano, saffron (laying hens)	0.5, 2	E	FUPerf	Daily FI, F:G	NS	NS	56 d	Botsoglou et al., 2005
Green tea (powder;extract, GTE) (laying hens), Marigold extract and powder	0.5, 1.5	E	GROPerf	WG	NS	NS	12 wk	Ariana et al., 2011
			FUPerf	FI	NS	↓6% (GTE)		
				F:G	NS	↓12.9% (GTE)		
Roselle Calyx powder and extract (laying hens)	1,2	E	FI, Feed/kg egg		NS	NS	8 weeks	Sukkhavanit et al., 2011
Anise seed (quails)	1,2	E	FUPerf	FI	NS	NS	29 d	Christaki et al., 2011
			Mortality		NS	NS		
Oregano and rosemary essential oils (OO, RO)	0.015% (OEO150); 0.03% OEO300); 0.015% (RO150); 0.03% (RO300) OEO and RO both at 0.0075% (OEO75+RO75); OEO and RO both at 0.015% (OEO150RO150)	E	GROPerf	BW (21 d)	↓4.7% (RO150)	↓4.8% (OEO150), ↓5.6% (RO150), ↓5.3% (OEO75RO75)	42 d	Basmacioglu et al., 2004
				BW (42 d)	NS	↓6.45% (OEO75RO75), ↓4.75% (OEO150RO150)		
				WG (0-21 d)	↓ 4.78% (RO150)	↓2.73% (OEO150), ↓3.33% (RO150), ↓3% (OEO75RO75)		

				WG (21-42 d)	NS	↓5.11% (OEO150), ↓6.96% (OEO75RO75), ↓5.97% (OEO150RO150)		
			FUPerf	FI	NS	NS		
				F:G	NS	NS		
			Mortality		NS	NS		
Oregano Plant (OP)	0.5% individually (OP); or with 190 mg/kg of Vitamin C (OC), or with 170 mg/kg (OE), or with both (OCE)	C, E, C+E	GROPerf	WG (28 d)	↑17.2% (OP), ↑18.3% (OC), ↑15.25% (OE), ↑19.2% (OCE)	NS	42 d	Florou-Paneri et al., 2006
				WG (35 d)	↑13.1% (O), ↑11.48% (OC), ↑10.59% (OE), ↑12.3% (OCE)	NS		
				WG (42d)	↑15.86% (O), ↑18.2% (OC), ↑14.9% (OE), ↑19.1% (OCE)	NS		
			FUPErf	F:G (28 d)	↓5.77% (O), ↓5.77% (OC), ↓5.13% (OE), ↓ 5.77% (OCE)	NS		
				F.G (35 d)	↓5.85% (O), ↓5.85% (OC), ↓5.85% (OCE)	NS		

				F:G (42 d)	↓7.73% (O), ↓8.25% (OC); ↓8.25% (OE), ↓8.25% (OCE)	NS		
Rosemary leaves (RL), rosehip fruits (RF), chokeberry pomace (CHKP), entire nettle (EN)	2.5	E	FUPerf	ADFI (Wk 2)	NS	NS	28 d	Loetscher et al., 2013
				ADFI (Wk 3)	NS	NS		
				ADFI (Wk 4)	↑17.86% (RF), ↑12.86% (CHKP)	↑8.55% (CHKP)		
				ADFI (Wk 5)	NS	NS		
				ADFI (Wk 2- 5)	NS	NS		
				F:G (Wk 2)	↑3.76% (RL)	↑3.76% (RL)		
			GROPerf	ADG (Wk 2)	↓ 11.1% (RL)	NS		
				ADG (Wk 3)	NS	NS		
				ADG (Wk 4)	↑19.44% (RF)	NS		
				ADG (Wk 5)	NS	NS		
				ADG (Wk 2-5)	NS	NS		
			DWE	DWE (Wk 2)	NS	NS		
				DWE (Wk3)	↓25.7% (RL)	NS		
			Mortality		NS	NS		
			AD and metabolizability of nutrients, energy, DM content of excreta	AD of NDF (Week 5)	↓50% (CHKP)	NS		

				AD of ADF (Wk 5)	NS	↓ 142.6% (RL), ↓126% P)		
				Metabolizability of nitrogen (Wk 5)	NS	NS		
				Metabolizability of energy (Wk 3)	↓5.12% (CHKP)	↓4.42% (RL), ↓5.98% (CHKP)		
				Metabolizability of energy (Wk 5)	↓6.68% (RL)	↓6.44% (RL)		
				ME (Wk 3)	↓4.64% (CHKP)	↓4.8% (RL), ↓5.9% (CHKP)		
Coneflower, Thyme, Sage, Marigold extracts,	0.056, 0.002 marigold xantophylls	E; BHT+EQ+BHA, (SYNT1); xantophyll, (SYNT2)	GROPerf	BW	NS	NS	42 d	Koreleski and Swiatkiewicz, 2007
			FUPerf	FI, F:G	NS	NS		
Olive leaf extract (OL)	0.02, 0.04	E	GROPerf	BW (1-42d)	NS	NS	14 d	Agah et al., 2019
			AND	AD of energy	↑11.% (0.02%), ↑2.71% (0.04%)	↓8.5 %(0.04%)		
				AD of crude protein	NS	↓14.47 %(0.04%)		
				AD of ash	↑53.26% (0.02%)	↓22.68 % (0.04%)		
				AD of Ca	↑44.81% (0.02%),	NS		
				AD of P	↑18.47% (0.02%)	↓ 15.65% (0.04%)		
Hesperidin	0.15, 0.3	E	GROPerf	WG, FBW	NS	NS	40 d	Simitzis et al., 2011
			FUPerf	F:G	NS	NS		
Forsythia suspensa extract	0.0001	C	GROPerf	ADG (22-42 d)	↑10.6%	↑4.26%	42 d	Wang et al., 2008
				ADG (1-42 d)	↑8.71%	↑4.17%		
			FUPerf	ADFI	NS	NS		
				F:G (22-42d)	↓6.34%	NS		
				F:G (1-42d)	↓4.37%	NS		

			Apparent ND	Energy (42 d)	↑3.87%	NS		
				Crude protein (42d)	↑3.91%	↑2.74%		
				Ca (42d)	↑10.1%	↑0.94%		
				P (42 d)	↑12.16%	NS		
Polyphenol product (PP)	0.01+100 mg/kg VitE(PPE); 0.02% (PP)	E, 100 mg/kg (100VE), 200 mg/kg (200VE)	GROPerf	BW (28 d)	TN:NS, HS: ↑16.1% (PPE), ↑20.2% (PP)	NS	35 d	Mazur-Kusnirek et al., 2019
				WG (28 d)	TN:↑6.45% (PP); HS: ↑16.77% (PPE), ↑21.1% (PP)	100VE: ↑5.64% (PP), 200VE: ↑3.51% (PP)		
			FUPerf	F:G	NS	NS		
				FI (28 d)	HS: ↑18.98% (PP)	NS		
Tomato skin, orange peel, green tea, leaves (ducks and broilers)	0.2	E	GROPerf	BW	NS	NS	20 d for broilers, 26 for ducks	Marzoni et al. 2014
				FBW	NS	NS		
				F:G	NS	NS		
Oregano aqueous extract (OaE)	0.02	E (150 mg/kg)	GROPerf	BW (1 d)	NS	NS	42	Forte et al. (2018)
				BW (21 d)	↑7.31%	↑8.88%		
				BW (42 d)	↑8.93%	NS		
				ADG (1-21d)	NS	NS		
				ADG (21-42d)	↑14.67%	NS		
			FUPerf	F:G	NS	NS		
Sage extract (SE)	2.5	E (30 mg/kg)	FUPerf	FI	NS	NS	28	Loetscher et al. (2014)
			GROPerf	BW change, FBW	NS	NS		
Thyme oil	0.01, 0.02	E, 100 mg/kg (E100), 200 mg/kg (E200)	GROperf	BW	↓0.58% (0.01%)	E100: ↑2.42% (0.01%) ↑3.15% (0.02%%) E200: ↓0.92% (0.01%)	42	Bölükbaşi et al. (2006)

				WG	↓0.6% (0.01%)	E100: ↑2.51% (0.01%) ↑3.3% (0.02%%) E200: ↓0.98% (0.01%)		
			FUPerf	FI	↑3.36% (0.01%) ↑4.04 % (0.02%%)	E100: NS E200: ↑1.7% (0.01%) ↑2.37 % (0.02%%)		
				F:G	↑1.71% (0.01%) ↑1.14% (0.02%%)	E100: ↓3.78% (0.01%) ↓4.32% (0.02%) E200: ↑2.89% (0.01%) ↑2.3% (0.02%%)		
Oregano oil, 2 different diets (CSB = crude soybean oil diet; ASO = acidulated soybean oil soapstock)	0.01	E, 10 mg/kg (E10), 100 mg/kg, (E100)	FUPerf	FC, F:G	NS	NS	42	Avila-Ramos et al. (2012)
			GROPerf	WG	NS	NS		
			Mortality		NS	NS		
Polyphenols	0.01+100 mg/kg of VitE (0.01E), 0.02,	E, 100 mg/kg, (E100), 200 mg/kg, (E200)<br>2 control, 1 negative (without low quality oil), 1 positive (with low quality oil)	FUPerf	FI, F:G	NS	NS	35	Mazur-Kusnirek et al. (2019)
			GROPerf	BW, BWG	NS	NS		
			Mortality		NS	NS		
			GROPerf	BW, BWG	NS	NS		
			Mortality		NS	NS		
Polyphenols	0.01+100 mg/kg of VitE (0.01E), 0.02,	E, 100 mg/kg, (E100); 200 mg/kg, (E200)	GROPerf	BW (d 7)	NS	NS		Mazur-Kusnirek et al. (2019)

		2 control, without (NG) or with grain (WG) contaminated						
				BW (d 21)	NG: ↓25.13% (0.01E) ↓27.4% (0.02%)	NS		
				BW (d 35)	NG: ↓16.82% (0.01E) ↓15.68% (0.02%)	NS		
				BWG	NG: ↓17.21% (0.01E) ↓16.1% (0.02%)	NS		
			FUPerf	F:G, FI	NS	NS		
			Mortality		NS	NS		
Rosemary (RVO), oregano (OVO) and fennel volatile oil (FVO)	0.01, equal mixture of each, VOM1 (0.01), VOM2 (0.02), VOM3 (0.03)	E, 200 mg/kg	GROPerf	BW (d 1)	NS	NS	42	Cetin et al. 2016
				BW (d 7)	↑5.88% (OVO) ↑5.25% (RVO) ↑8.88% (FVO) ↑5.93% (VOM2) ↑4.99% (VOM3)	↓5.87% (VOM1)		

	BW (14 d)	↑8.48% (OVO)<br>↑7.41% (RVO)<br>↑11.25% (FVO)<br>↑8.62% (VOM2)<br>↑9.53% (VOM3)	↓7.62% (VOM1)
	BW (d 21)	↑6.27% (OVO)<br>↑6.48% (RVO)<br>↑8.56% (FVO)<br>↑6.15% (VOM2)<br>↑8.23% (VOM3)	↓8.43% (VOM1)
	BW (d 28)	NS	NS
	BW (d 35)	↑7.62% (VOM3)	NS
	BW (d 42)	↑7.57% (VOM3)	NS
	BWG (1.42 d)	↑7.73% (VOM3)	NS
FUPerf	FI	NS	NS
	F:G	↓8.33% (VOM3)	↓7.82% (VOM3)

Abbreviations: AA, antioxidant activity; AD, apparent digestibility; ADFI, average daily feed intake; ADG, average daily gain; AFW, average final weight; AIDEA, apparent ileal digestibility of essential aminoacids; AIDF, apparent ileal digestibility of fat; AIDP, apparent ileal digestibility of proteins; BW, body weight; DWE, daily water expenditure; EPEF, European production efficiency factor; FBW, final body weight; FC, feed consumption; FDCT, faecal digestibility of condensed tannins; FDHP, fecal digestibility of utilizable polyphenols; FE, feed efficiency; F:G, feed to gain ratio; FI, feed intake; FUPerf, feed utilization performances; GROPErf, growth performances; HS, heat stressed group; IDCT, ilea digestibility of condensed tannins; IDHP, ileal digestibility of hydrolizable polyphenols; ND, nutrient digestibility; NS, not significative; PFA, plant feed additive;; TEP = total extractable polyphenol; TICT, total intake of condensed tannins; TIHP, total intake of hydrolizable polyphenols; TN, thermoneutral reared group; WG, weight gain.

Table S2. Effects of different plant feed additives on metabolic, haematological, immunological, oxidative stress parameters in poultry.

PFA	Dose extract (% of the diet, as FED)	Vitamin of comparison	Class of parameters	Traits evaluated	Comparison to negative control: effect (% of variation, PFA dose)	Comparison to positive control: effect (% of variation, PFA dose)	Period of study (days, d; weeks, wk)	Reference
Grape pomace	5, 7.5, 10	E, 200 mg/kg	Biochemical parameters	HDL-C	↑50.9% (10%)	↑49.3% (10%)	42 d	Ebrahimzadeh et al. (2018)
				LDL-C	between ↓26.7% and ↓36.7% (10%)	↓28.6% (10%)		
				TG	↓from 34.7% to ↓42%	NS		
				AST	↑72% (10%)	↑ 55.1%		
			AS markers	SOD	↑55.9% (7.5%), ↑42.8% (5%)	↑45.6% (7.5%)		
				GPx	↑46.3% (5%), ↑59.02% (7.5%)	↑ 33.75% (7.5%)		
				MDA	↓ 38.4% (7.5%)	↓12% (7.5%)		
			Antibody titers	NDV antibody titer 42d	↑ 43.18% (5%), ↑ 40.91% (10%)	↓23.3%		
				SRBC antibody titer				
				Primary titer	NS	NS		
				IgG	NS	NS		
				IgM	↑60% (7.5%), ↑ 80%(10%)	50% (10%)		
				Secondary titer				
				IgG	↑350% (10%)	↓-57.9% (5%)		

				IgM	NS	NS		
Grape pomace	1.5 , 3, 6	E	AAEP of ileal content, excreta and serum	Diet antioxidant activity (ABTS)	↑ between + 225% and 552% (1.5, 3 and 6%)	↑ between 64.6% and 230.4% (1.5, 3 and 6%)	21 d	Brenes et al. (2008)
				Ileal antioxidant activity (ABTS)	↑ between 55.7% and 175% (1.5, 3 and 6%)	↑ between 32.4% and 104% (1.5, 3 and 6%)		
				Excreta antioxidant activity (ABTS)	↑ between 39.6% and 43.5% (1.5, 3 and 6%)	NS		
				Serum antioxidant activity (ABTS)	NS	NS		
				Diet antioxidant activity (FRAP)	↑ from 145.5% to 445.5% (1.5, 3 and 6%)	↑ between +102.5% and 350% (1.5, 3 and 6%)		
				Excreta antioxidant activity	NS	NS		
				Ileal antioxidant activity (FRAP)	NS	NS		
Grape pomace	0.5, 1.5, 3	E	Total intake (only 3 % of the diet) and vitamin E	Total intake of extractable polyphenols (g)	↑ 60.1% (3%)	↑ 41.1% (3%)	21 d	Goñi et al. (2007)
			Digestibility	Digestibility of extractable polyphenols	↑ 129.4 % (3%)	↑ 105.0% (3%)		
			AAEP in diets	Diet (ABTS)	NS	NS		
			AAEP in excreta	Excreta (ABTS)	↑ 19.9% (3%)	NS		
			AAEP in excreta	Excreta (FRAP)	↑ 18.8% (3%)	NS (3%)		
			AAEP in serum	Serum (ABTS)	NS	NS		
Grapeseed extract	0.015, 0.03, 0.045	C (300 mg/kg)	SG, TG, LDL in serum blood at day 28 and day 42	Glucose (28)	↓ from 13% to 10.5%	↓ from 7.0 % to 9.7%	From 1 day to 42 day	Hajati et al. (2015)
				Glucose (42)	↓ from 10.8% to 20.8%	↓ from 6.8% to 17.2%		
				TG (28)	NS	NS		

				TG (42)	↓10.8% (0.045%)	NS		
				CHOL (28)	NS	NS		
				CHOL (42)	↓12.8% (0.045%), ↓5.1% (0.030%)	↓ 5.94% (0.045%) ↑6.2% (0.015%)		
				HDL-C (28)	NS	NS		
				HDL-C (42)	NS	NS		
				LDL-C (42)	↓ 27.0 % (0.045%)	NS		
				VLDL (28)	NS	NS		
				VLDL (42)	↓ 10.7 % (0.045%)	NS		
				UA (28)	NS	NS		
				UA (42)	NS	NS		
			HSP70 gene expression in heart	ΔCt° (gene expression, 42 d)	↑ from 425% to 686%	NS		
			HSP70 gene expression in liver	ΔCt° (gene expression 42 d)	↑ from 173.8% to +240%	↑ 21.7%		
Grape polyphenols	0.0025, 0.0050, 0.0075	E	Antibody titers against NDV and IBD	NDV		*↑ 51.24% (0.025% of GPP+ 75 mg/kg of VitE), ↑59.2% (0.075% of GPP+ 25 mg/kg of VitE)	From day 1 to 35	Iqbal et al. (2015)
				IBD		↑ 597.2% (0.075% of GPP+25 mg/kg of VitE, ↑ 429% (comp to LGPP), ↑ 349.2% (comp to MGPP)		

Clove bud powder (Syzygium aromaticum) with two n-6 to n-3 ratios (16.71 and 2.35) laying hens	0.2, 0.4	E, two groups: low fatti acid diet (LFA) and high fatty acid diet (HFA)	Biochemical parameters	CHOL	HFA : ↓ 34.8% (0.4%); LFA: ↓21% (0.2%), 30.6% (0.4%)	HFA: ↓ 21% (0.4%); LFA: ↓ 10.5% (0.2%), 21.4% (0.4%)	70 d	Rahman Alizadeh et al. (2017)
				TG	HFA: ↓ 20.4% (0.2%); ↓ 39.6% (0.4%); LFA: ↓from 22.5% to 35%	LFA: ↓ from 9.7% to 16.8%		
				HDL	HFA: ↑40% (0.2%) ↑73.1% (0.4%); LFA diet:↑25.5% (0.2%), ↑54.2% (0.4%)	HFA: ↓13.6% (0.2%) ↑6.78% (0.4%); LFA: ↓4,9% (0.2%), ↑16.9% (0.4%)		
				LDH	NS	NS		
				AST	HFA: ↓41.7% (0.2%) ↓68% (0.4%); LFA: ↓54.64% (0.2%), ↓81.4% (0.4%)	HFA:↓3.45% (0.2%), ↓47.13% (0.4%); LFA: ↓25.4% (0.2%), ↓69.5% (0.4%)		
				ALT	HFA: ↓ from 10.3% (0.2%) to 31.4% (0,4%); LFA: ↓12.6% (0.2%) ↓33.2%% (0.4%	HFA: ↓27.3% (0.4%); LFA: ↓ 28.95% (0.4%)		
				ALP	NS	NS		
			Antioxidant activity	MDA in blood				
				Middle period (47 weeks)	NS	NS		

				Final period (52 weeks)	HFA: ↓45.1% (0.4%); LFA: ↓ 25.6% (0.2%) ↓40.9% (0.4%)	HFA: ↓19.2%, LFA: ↓11.2% (0.4%)		
				TAC	HFA: ↑37.5% (0.2%) ↑45.4% (0.4%): LFA: ↑ 80.98% (0.4%)	HFA: ↑13.8% (0.2%) ↑20.3% (0.4%); LFA: ↑ 5.99% (0.2%) ↑21% (0.4%)		
				VitE level	HFA: ↑50% (0.2%) ↑109.3% (0.4%); LFA: ↑103.1% (0.4%)	HFA: ↓81.1% (0.2%) ↓73.6% (0.4%); LFA: ↓84.4% (0.2%) ↓77.2% (0.4%)		
Rosemary plant and Rosemary oil	0.57%, 0.86%, 1.15%;	E	Antioxidant activity	SOD activity	NS	NS	42 d	Yesilbag et al. (2011)
Rosemary powder (RP)	0.5, 1.0	E	Immune response after vaccination protocol	Total antibody titer			42 d	Rostami et al. (2018)
			Total antibody		NS	NS		
			TP		NS	NS		
			Biochemical	Globulin	Only significant RP x VE interaction	Only significant RP x VE interaction		
			parameters					
				Albumin	NS	NS		

Green tea extract (GTE) and powder (GTP), Marigold extract (ME) and powder (MP)	0.5 (GTP,ME), 1.5% (GTE, MP) laying hens	E (200 mg)	Biochemical parameters	CHOL	↓7.9% (MP) ↓ 7.4% (ME) ↓10.3% (GTE)	NS	12 wk	Ariana et al. (2011)
				HDL/CHOL	↑9.7% (ME), ↑7.98% (GTE)	NS		
				TG	↓ 7.32 % (GTE)	NS		
				HDL/LDL	↑12.4% (MP) ↑15.5% (ME) ↑14.4% (GTP) ↑17.5% (GTE)	NS		
				GL	NS	NS		
Roselle Calyx	Crude extracts (1,2%), Powder (2,4%), laying hens	E	TBARs in plasma	After 4 week	NS	NS	8 wk	Sukkhavanit et al. (2011)
				After 8 week	NS	NS		
Anise seed (quails)	1%, 2%	E (600 mg/kg)	Biochemicalparameters	Tot CHOL	↓ 23.5% (1%), only tendency, *P*=0.077	↓ 23.3% (1%)	29 d	Cristaki et al. (2011)
				TG	NS	NS		
Olive leaf extract (OL), broilers	0.2% (OL1), 0.4% (OL2)	E	GLU, CHO, TG, UA, TP, Serum enzymes, FRAP, SOD, GPx, MDA in plasma	GLU	NS	NS	14 d	Agah et al. (2019)
				CHOL	↓ 15,5% (0.2%) ↓9.5% (0.4%)	NS		

				TG	↓ 19.5% (0.2%) ↓34% (0.4%)	NS		
				UA	↓28% (0.2%) ↓47.7% (0.4%)	↓ 30,3% (0.4%)		
				TP	NS	NS		
			Serum enzymes					
				AST	NS	NS		
				ALT	↓ 18% (0.4%)	NS		
				ALP	↓8.1% (0.2%), ↓20,5% (0.4%)	↓ 15,7% (0.4%)		
				FRAP	↑ 26,5% (0.4%) ↑40.9% (0.2%)	↓ 19,1% (0.4%)		
				SOD	NS	NS		
				GPx	↑ 27% (0.2%) ↑21.2% (0.4%)	NS		
				Plasma MDA	↓13.1% (0.2%) ↓ 28,2 % (0.4%)	↓ 16,9% (0.4%)		
Sweet chestnut wood extract (SCW), broilers	0.3%, or in combination with 68 UI of VitE (SCWE): 2 controls: CP (C-P) CLIN (C-Lin):	E, 85 mg/kg(E85) and 200 mg/kg (E200)	Mitigating effect on DNA damage of high PUFA diets; Lymphocyte DNA damage (OTM), Tail DNA (%), Plasma MDA, Liver MDA, TAS, ACL, SOD, GPx, GR	OTM	CLIN: ↓42.4% (0.3%), ↓41.3% (SCWE)	NS	24 d	Voljc et al. (2013)
				Tail DNA (%)	CLIN: ↓25.3% (SCWE)	NS		

	Plasma MDA	CP: ↑261.5 % (0.3%) CLIN:↓43.1% (SCWE)	NS
	Liver MDA	CP: ↑73.6% (0.3%) ↑81.1% (SCWE)	NS
	TAS	NS	NS
	SOD	NS	NS
	ACL	CLIN: ↑22.2% (SCWE)	VitE 200 mg/kg: ↓16.7% (0.3)
	GPx	NS	NS
	GR	NS	NS
Alfa, beta, gamma and total tocopherols in plasma	α	CP: ↑173.9% (SCWE) CLIN: ↑316.6% (SCWE)	VitE 85mg/kg: ↓69.1% % (0.3), VitE200: ↓38.4%
	β + γ	CP: ↑ 196.4% (0.3%) CLIN: ↑31.5% (0.3%)	VitE85: ↑ 114.5% (0.3%) VitE200: ↑ 117.3% (0.3%)
	Tot tocopherols	CP: ↑ 165.8% (SCWE) CLIN: ↑ 242% (SCWE)	VitE85: ↓61.8% (0.3%); VitE200 ↓79.7% (0.3%), ↓36.9% (SCWE)
In liver	α	CP: ↑250.9% (SCWE); CLIN: ↑339.2% (SCWE)	VitE85: ↓70.96% (0.3%); VitE200: ↓85.7% (0.3%) ↓39.6% (SCWE)

				β + γ	CP: ↑174.2% (SCW) ↑112.9% (SCWE)	VitE85: ↑ 60.4% (0.3%)		
				Tot tocopherols	CP: ↑236% (SCWE); CLIN: ↑254.9% (SCWE)	VitE85: ↓61.7% (0.3%),↑22.3% (SCWE); VitE200: ↓80.6% (0.3%) ↓38.3% (SCWE)		
			In breast muscle	α	CP: ↑154.4% (SCWE); CLIN: ↑240.6% (SCWE)	VitE85: ↓58.96% (0.3%), ↑28% (SCWE); VitE200: ↓80.31% (0.3%), ↓38.6% (SCWE)		
				β + γ	CP: ↑152.2% (0.3%) ↑154.4% (SCWE); CLIN: NS	VitE85: ↓39.8% (0.3%); VitE200: ↑41.5% (0.3%)		
				Tot tocopherols	CP: ↑148.9% (SCWE); CLIN: ↑170% (SCWE)	VitE85: ↓49.2% (0.3%) ↑26.4% (SCWE); VitE200 ↓74.3% (0.3%), ↓35.9% (SCWE)		
Natural tocopherols (NT), rosemary, grape seed (GSE), green tea (GT), tomato alone (TO), or in different combination	0.01, 0.02% and different combinations	Synthetic AntioxidAnts (SYN) And E	Frap, SOD, TBARS	No control without " vitamin	NS (only a trend for SOD)	NS (only a trend for SOD)	42 d	Vossen et al. (2011)
			GPx	No control without " vitamin		↑ from 23.6% (0.01% of RO) to 100.6% (0.02% of TO)		

Polyphenol product (PP)	0.01% along with 100 mg of VitE (PPE) and 0.02% (PP)	E, 100 mg/kg(E100), 200 mg/kg(E200)	AS in blood and tissues in HS broilers and TN reared broilers	Blood			35 d	Mazur-Kusnirek et al. (2019c)
			Blood	TAS	NS	NS		
				SOD	TN: ↓18.3% (PPE) ↓21.8% (PP); HS: NS	NS		
				GPx	TN: NS; HS: ↑49.1% (PPE)	NS		
				Retinol	TN: ↓21.9% (PPE) ↓25% (PP); HS: NS	NS		
				Total TOC	TN: ↓41.1% (PPE); HS:↑195.9% (PPE) ↑261.5% (PP)	VitE100: ↓45.9% (PPE) ↓33.9% (PP); VitE200: ↓53.2% (PPE) ↓42.85 (PP)		
				VitE equivalent	TN: ↓55.8% (PPE) ↓35.3% (PP); HS: ↑500% (PP)	VitE100: ↓62.6% (PPE) ↓45.2% (PP); ViE200: ↓67.5% (PPE) ↓52.3% (PP)		
			Liver	VitC	NS	NS		
				Retinol	TN: ↓14.5% (PPE) ↓19.3% (PP); HS: ↓6.7% (PP)	VitE100: ↓14.3% (PPE) ↓19.2% (PP): VitE200: ↓7.2% (PP)		

	Total TOC	TN: ↑248.3% (PPE) ↑183.7% (PP); HS: ↑409.2% (PPE) ↑314.8% (PP)	VitE100: ↑195.8% (PPE) ↑141% (PP); VitE200: ↓31.5% (PP), ↓44.2% (PPE)
	VitEEq	TN: ↑396.7% (PPE) ↑299% (PP); HS: ↑697.4% (PPE) ↑540.6% (PP)	VitE100: ↑294.7% (PPE) ↑217.1% (PP); VitE200: ↓31.9% (PPE) ↓45.3% (PP)
	TBARS	TN: ↑32.2% (PPE) ↑31.9% (PP)	NS
Breast	Vitamin C	TN: ↓12.8% (PP); HS: NS	NS
	Retinol	TN: ↓88.6% (PPE) ↓88.5% (PP); HS: NS	NS
	Total TOC	TN: ↑143.3% (PPE) ↑160.9% (PP); HS: ↑216.9% (PPE) ↑239.8% (PP)	VitE100: NS; VitE200: ↓29.2% (PPE) ↓24.1% (PP)
	VitEEq	TN: ↑248% (PPE) ↑280% (PP); HS: ↑383% (PPE) ↑427.9% (PP)	VitE100:↓15.5% (PPE); VitE200: ↓26.4 % (PP)
	TBARS	TN: NS; HS; NS	NS

Thyme oil	0.01, 0.02	E, 100 mg/kg (E100),200 mg/kg (E200)	Biochemical parameters	Tot CHOL	↑3.01% (0.02%)	E100: ↑7.1% (0.01%) ↑10.32% (0.02%) E200: ↓3.49% (0.01%)	42	Bölükbaşi et al. (2006)
				TG	↑34.07% (0.01%) ↑48.35% (0.02%)	E100: ↑154.43% (0.01%) ↑70.89% (0.02%) E200: ↑25.77% (0.01%) ↑39.18% (0.02%)		
				HDL-C	↑20.88% (0.01%) ↑6.59% (0.02%)	E100: ↑8.91% (0.01%) ↓3.96% (0.02%) E200: ↓4.35% (0.01%) ↓15.65% (0.02%)		
				LDL-C	↑18.27% (0.01%) ↑20.19% (0.02%)	E100: ↑23.74% (0.01%) ↑25.76% (0.02%) E200: ↑6.03% (0.01%) ↑7.76% (0.02%)		
Polyphenols	0.01+100 mg/kg of VitE (0.01E), 0.02	2 control, 1 negative (NO, without low quality oil), 1 positive (WO)with low quality oil					35	Mazur-Kusnirek et al. (2019b)
			Antioxidant status blood	TAS, SOD	NS	NS		

	GPx	WO: ↑20.47% (0.01E) ↑11.88% (0.02%)	E100: ↑14.98% (0.01E) ↑14.25% (0.02%)
	Retinol	NO:↑45.33% (0.02%) WO: ↑45.33% (0.02%)	E100: ↑53.52% (0.02%) E200: ↑34.57% (0.02%)
	Total tocopherols	NO:↑338.67% (0.01E) WO: ↑416.75% (0.01E) ↑48.69% (0.02%)	E100: ↑36.14% (0.01E) ↓60.83% (0.02%) E200: ↓24.89% (0.01E) ↓78.39% (0.02%)
	Vitamin EEq	NO: ↑380.69% (0.01E) =0.02% WO: ↑477.98% (0.01E) =0.02%	E100: ↑36.57% (0.01E) ↓64.56% (0.02%) E200: ↓64.57 (0.01E) ↓80.6% (0.02%)
Antioxidant status	Vitamin C	WO: ↑10.08% (0.01E) ↑8.64% (0.02%)	NS
	Retinol	NO: ↓36.16% (0.01E) ↓11.75% (0.02%) WO: ↑72.31% (0.01E) ↑138.21% (0.02%)	E100: ↑28.92% (0.01E) ↑78.22% (0.02%); E200: ↑89.38% (0.01E) ↑161.8% (0.02%)

			Total tocopherols	NO: ↑204.36% (0.01E) WO: ↑865.19% (0.01E) ↑130.94% (0.02%)	E100: ↑73.14% (0.01E) ↓58.57 % (0.02%) E200: ↑26.23% (0.01E) ↓69.80% (0.02%)		
			Vitamin EEq	NO: ↑394.75% (0.01E) ↓31.48% (0.02%) WO: ↑873.55% (0.01E) =0.02%	E100: ↑52.73% (0.01E) ↓78.85% (0.02%) E200: ↑9.83% (0.01E) ↓84.79% (0.02%)		
			TBARS	NS	NS		
Polyphenols	0.01+100 mg/kg of VitE (0.01E), 0.02,		2 control, without (NG) or with grain (WG) contaminated			35	Mazur-Kusnirek et al. (2019a)
		Antioxidant status blood	TAS	WG: ↑23.53% (0.01E) ↑21.57% (0.02%)	NS		
			SOD	NG: ↓18.42% (0.02%) WG: NS	E100: ↑10.65% (0.01E) E200: ↓12.83% (0.02%)		
			GPx	NG: NS WG: ↑17.56% (0.01E) ↑34.59% (0.02%)	NS		
			Retinol	NG: ↓66.95% (0.01E) ↓72.88% (0.02%) WG: NS	E100: NS E200: ↓49.35% (0.01E) ↓58.44% (0.02%)		

	Total tocopherols	NG: ↑31.5% (0.01E) WG: ↑632.65% (0.01E) ↑365.31% (0.02%)	E100: ↑37.02% (0.01E) E200: ↓25.21% (0.01E) ↓52.5% (0.02%)
	Vitamin E equivalent	NG: ↑52.21% (0.01E) WG: ↑1538.1% (0.01E) ↑928.57% (0.02%)	E100: ↑46.38% (0.01E) E200: ↓26.34% (0.01E) ↓53.75% (0.02%)
Antioxidant status Liver	Vitamin C	NS	E200: ↓17.28% (0.01E) ↓16.87% (0.02%)
	Retinol	NG: ↓14.16% (0.01E) ↓15.27% (0.02%)	E100: ↑29.77% (0.01E) ↑28.09% (0.02%) E200: ↑20.5% (0.01E) ↑18.94% (0.02%)
	Total tocopherols	NG: ↑109.4% (0.01E) WG: ↑150.26% (0.01E)	E100: ↑31.49% (0.01E) ↓45.52% (0.02%) E200: ↓28.69% (0.01E) ↓70.46% (0.02%)

	VitE equivalent	NG: ↑137.7% (0.01E) WG: ↑392.19% (0.01E)	E100: ↑38.93% (0.01E) ↓51.73% (0.02%) E200: ↓30.32% (0.01E) ↓75.79% (0.02%)
	TBARS	WG:↓37.16% (0.01E)	NS

Abbreviations: AAEP, antioxidant activity of extractable polyphenols; ABTS, 2,2'-azino-bis(3-ethylbenzothiazoline-6-sulfonic acid; ACL, antioxidant capacity of lipid-soluble antioxidants; ALP, alkaline phosphatase; ALT, alanine aminotransferase; AS, antioxidant status; AST, aspartate aminotransferase; CHOL, cholesterol; CLIN, control linseed oil enriched diet; CP, palm fat enriched diet; FRAP, ferric reducing activity power; GLU, glucose; GPx, glutathione peroxidase; GR, glutathione reductase; HDL-C, high density lipoproteins cholesterol; HFA, high n6 to n3 fatty acids ratio; HS, heat stresses group; IBD, infectious bursal disease; LDH, lactate dehydrogenase; LDL-C, low density lipoproteins cholesterol; LFA, low n-6 to n-3 fatty acids ratio; MDA, malondialdehyde; ; NDV, Newcastle disease virus; NS, not significative; OTM, olive tail moment; PFA, plant feed additive; SG, serum glucose; SOD, superoxide dismutase; SRBC, sheep red blood cells; TAC, total antioxidant capacity; TAS, total antioxidant status; TBARS, thiobarbituric acid reactive substances; TG, triglyceride; TN, thermoneutral reared group; TOC, tocopherols; TP, total proteins; UA, uric acid; VLDL, very low density lipoproteins.

Table S3. Effects of plant feed additives on gastrointestinal anatomic features and microbiota in poultry.

PFA	Dose extract (% of the diet, as FED)	Vitamin of comparison	Class of parameters	Traits evaluated	Comparison to negative control: effect (% of variation, PFA dose)	Comparison to positive control: effect (% of variation, PFA dose)	Period of study (days, d; weeks, wk)	Reference
Oregano aqueous extract	0.02	E	Gut complex carbohydrates	Goblet cell reactivity	↑	↑	42 d	Scocco et al. (2017)
			Intestinal microflora count in ileum	Coliform (21 d)	↓19.31%	↓22.35%		
				Coliform (d 42)	↓24.1%	↓23.43%		
				E. coli (21)	↓15.72%	↓27.17%		
				E. coli (42)	NS	↓20.58%		
				Staphylococcus spp (21)	NS	NS		
				Staphylococcus spp (42)	NS	NS		
				Enterococcus (21)	NS	NS		
				Enterococcus (42)	NS	NS		
				LAB (21)	↓21.65%	↓19.96%		
				LAB (42)	NS	NS		
				Total anaerobia (21)	↓24.92%	↓20.12%		
				Total anaerobia (42)	↑18.99%	↑17.68%		
			Intestinal microflora count in caecum	Coliform (21)	NS	↓22.45%		
				Coliform (42)	NS	NS		
				E. coli (21)	↓17.57%	↓35.81%		
				E. coli (42)	↓9.46%	↓9%		
				Total anaerobia (21)	NS	↑8.22%		
				Total anaerobia (42)	↑18.53%	↑11.74%		
Grape pomace	5, 7.5, 10	E	Gut morphology	VH, CD, duodenum	NS	NS	42 d	Ebrahimzadeh et al. (2018)

				VH/CD, duodenum	↓27.75% (10%)	↑35.54% (5%)		
				MT duodenum	↓48% (5%) ↓25.98% (7.5%) ↓26.43% (10%)	NS		
				VH jejunum	↓33.32% (5%) ↓35.83% (7.5%) ↓41.54% (10%)	↓35.35% (10%)		
				CD jejunum	NS	↓38.58% (5%) ↓27.27% (10%)		
				VH/CD jejunum	↓37.69% (7.5%) ↓32.66% (10%)	NS		
				Muscularis thickness jejunum	NS	NS		
				VH, CD, VH/CD ileum	NS	NS		
				Muscularis thickness ileum	↓36.69% (5%) ↓37.98% (7.5%) ↓34.87% (10%)	NS		
Grape polyphenols	0.0025, 0.0050, 0.0075	E	Histopathological examination of kidney and liver tissues		NS	NS	35 d	Iqbal et al. (2015)
Grape pomace	1.5, 3, 6	E	Organ weights and lengths	RW liver, pancreas	NS	NS	21 d	Brenes et al. (2008)
				RW of AF	↑18.42% (1.5%)	NS		
				RW of spleen	↑20% (1.5%)	NS		
				RLE duodenum	↑7.41% (1.5%)	NS		
				Relative length of jejunum, ileum	NS	NS		
				Relative length of ceca	↑10.39% (1.5%)	NS		
Rosemary powder	0.5, 1.0	E	Lymphoid organs evaluation	Weight and RW of Thymus, Spleen, Bursa of Fabricius	NS	NS	42 d	Rostami et al. (2018)
Clove bud powder	0.2 , 0.4	E	Hepatic histological alterations	TI	HFA: ↑25% (0.2%) ↑58.3% (0.4%); LFA: ↑75% (0.2%) ↑50% (0.4%)	HFA: ↑25% (0.2%) ↑58.3 (0.4%); LFA: ↑47.37% (0.2%) ↑26.32% (0.4%)	70 wk	Rahman Alizadeh et al. (2017)
				KCN	NS	NS		
				FVN	HFA:↓34.62% (0.2%) ↓38.46% (0.4%) LFA: ↓25.93% (0.2%) ↓51.85% (0.4%)	HFA: ↑ 112.5% (0.2%) ↑100% (0.4%); LFA:↓35% (0.4%)		

Fermented (FGS) and unfermented (UGS) grape skin (GS)	3, 6	E	LAB, E. *coli* and *Clostridium* spp. in the ileal contents of 21-d broiler chickens	Count	NS	NS	21 d	Nardoia et al. (2019)

Abbreviations: AF, abdominal fat; CD, crypt depth; HTI, hepatic tissue integrity; FVN: fat vacuole number score KCN: Kupffer cell number score; HFA,high n-6 to n-3 fatty acids ratio diet; LFA, low n-6 to n-3 fatty acids ratio diet; MT, muscularis thickness; RLE, relative length; RW, relative weight; TI, tissue integrity; VL: villus length, V/C: ratio villus length/cry VL: villus length, V/C: ratio villus length/crypt.
